# A luminal EF-hand mutation in STIM1 in mice causes the clinical hallmarks of tubular aggregate myopathy

**DOI:** 10.1242/dmm.041111

**Published:** 2019-12-03

**Authors:** Celia Cordero-Sanchez, Beatrice Riva, Simone Reano, Nausicaa Clemente, Ivan Zaggia, Federico A. Ruffinatti, Alberto Potenzieri, Tracey Pirali, Salvatore Raffa, Sabina Sangaletti, Mario P. Colombo, Alessandra Bertoni, Matteo Garibaldi, Nicoletta Filigheddu, Armando A. Genazzani

**Affiliations:** 1 Department of Pharmaceutical Sciences, University of Piemonte Orientale, Via Bovio 6, Novara 28100, Italy; 2 Department of Translational Medicine, Università del Piemonte Orientale, Via Solaroli 17, Novara 28100, Italy; 3 Laboratory of Ultrastructural Pathology, Department of Clinical and Molecular Medicine, SAPIENZA University of Rome, Sant'Andrea Hospital, Rome 00189, Italy; 4 Department of Experimental Oncology and Molecular Medicine, Fondazione IRCCS Istituto Nazionale Tumori, Milan 20133, Italy; 5 Unit of Neuromuscular Disorders, Department of Neuroscience, Mental Health and Sensory Organs (NESMOS), Sapienza University of Rome, Sant'Andrea Hospital, Rome 00189, Italy

**Keywords:** STIM1, Calcium signaling, Mouse model, Rare disease, Store-operated calcium entry

## Abstract

STIM and ORAI proteins play a fundamental role in calcium signaling, allowing for calcium influx through the plasma membrane upon depletion of intracellular stores, in a process known as store-operated Ca^2+^ entry. Point mutations that lead to gain-of-function activity of either STIM1 or ORAI1 are responsible for a cluster of ultra-rare syndromes characterized by motor disturbances and platelet dysfunction. The prevalence of these disorders is at present unknown. In this study, we describe the generation and characterization of a knock-in mouse model (KI*-*STIM1^I115F^) that bears a clinically relevant mutation located in one of the two calcium-sensing EF-hand motifs of STIM1. The mouse colony is viable and fertile. Myotubes from these mice show an increased store-operated Ca^2+^ entry, as predicted. This most likely causes the dystrophic muscle phenotype observed, which worsens with age. Such histological features are not accompanied by a significant increase in creatine kinase. However, animals have significantly worse performance in rotarod and treadmill tests, showing increased susceptibility to fatigue, in analogy to the human disease. The mice also show increased bleeding time and thrombocytopenia, as well as an unexpected defect in the myeloid lineage and in natural killer cells. The present model, together with recently described models bearing the R304W mutation (located on the coiled-coil domain in the cytosolic side of STIM1), represents an ideal platform to characterize the disorder and test therapeutic strategies for patients with STIM1 mutations, currently without therapeutic solutions.

This article has an associated First Person interview with Celia Cordero-Sanchez, co-first author of the paper.

## Abstract (precis)

**Summary:** We describe a mouse model (KI*-*STIM1^I115F^) that displays the clinical hallmarks of tubular aggregate myopathy. This model provides a new opportunity to characterize the disorder and test novel therapeutic strategies.

## INTRODUCTION

High concentrations of calcium ions are present in intracellular organelles [in particular in the endoplasmic reticulum (ER)/sarcoplasmic reticulum (SR)], and the opening of Ca^2+^ channels located on these membranes (e.g. ryanodine receptors, inositol 1,4,5-trisphosphate receptors) allows this ion to flux out of the deposit and elicit cellular signals. A crosstalk mechanism between the ER and the plasma membrane exists that allows for the refilling of the depleted organelles (Putney, 2011). This crosstalk is known as store-operated Ca^2+^ entry (SOCE). The principal components of SOCE are a Ca^2+^ sensor on the ER membrane (STIM protein) and a plasma membrane Ca^2+^ channel (ORAI protein) (Lacruz and Feske, 2015; Berna-Erro et al., 2012). STIM proteins are single-span membrane proteins, highly conserved across species. Two members of the family have been described, STIM1 and STIM2, of which the former appears more expressed. ORAI channels reside on the plasma membrane, and three members of the family (ORAI1, ORAI2 and ORAI3) have been described, with ORAI1 being the most abundant. Importantly, other crucial proteins participate in the SOCE process, including transient receptor potential canonical (TRPC) channels (Ong and Ambudkar, 2015).

**Fig. 1.**
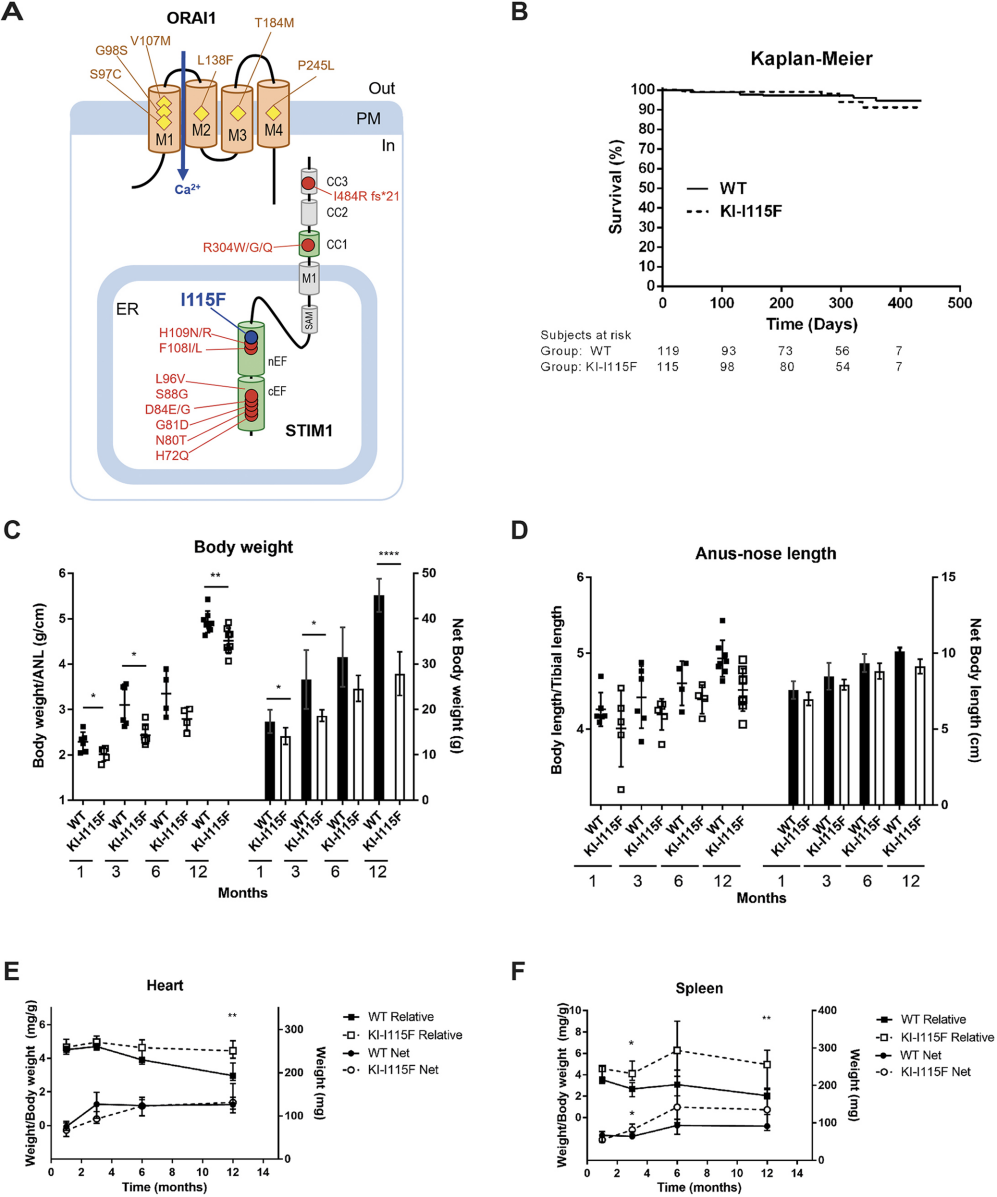
**Characterization of KI*-*STIM1^I115F^ mice multi-systemic phenotype.** (A) Known point mutations leading to gain-of-function phenotypes in humans. ER, endoplasmic reticulum; PM, plasma membrane. (B) Kaplan–Meier for survival of WT and KI-I115F. Numbers below the graph indicate numbers at risk, i.e. the number of true observations made to estimate survival. (C) Body weight at 1 (WT *n*=6, KI-I115F *n*=5), 3 (WT *n*=6, KI-I115F *n*=6), 6 (WT *n*=4, KI-I115F *n*=4) and 12 (WT *n*=8, KI-I115F *n*=8) months, shown as ratio to anal-nose length (ANL) on the left axis and net body weight on the right axis. Scatter plots and histograms show the means±s.e.m. of the indicated number of mice. Unpaired Student’s *t*-test with Welch's correction. *P≤0.0386, **P=0.0068, ****P<10^−4^ versus WT. (D) Body length at 1 (WT *n*=6, KI-I115F *n*=5), 3 (WT *n*=6, KI-I115F *n*=6), 6 (WT *n*=4, KI-I115F *n*=4) and 12 (WT *n*=8, KI-I115F *n*=8) months, shown as ratio to tibial length on the left axis and net body length on the right axis. Scatter plots and histograms show the means±s.e.m. of the indicated number of mice. Unpaired Student’s *t*-test with Welch's correction. (E) Heart weight at 1 (WT *n*=6, KI-I115F *n*=5), 3 (WT *n*=6, KI-I115F *n*=6), 6 (WT *n*=4, KI-I115F *n*=4) and 12 (WT *n*=8, KI-I115F *n*=8) months, shown as ratio to body weight on the left axis and net weight on the right axis. Graph shows median and IQR of heart weight/body weight, or heart net weight. Mann–Whitney *U*-test. **P=0.003 versus WT. (F) Spleen weight at 1 (WT *n*=6, KI-I115F *n*=5), 3 (WT *n*=6, KI-I115F *n*=6), 6 (WT *n*=4, KI-I115F *n*=4) and 12 (WT *n*=8, KI-I115F *n*=8) months, shown as ratio to body weight on the left axis and net weight on the right axis. Graph shows median and IQR of spleen weight/body weight, or spleen net weight. Mann–Whitney *U*-test. *P≤0.026, **P=0.0037 versus WT.

Genetic defects of STIM and ORAI proteins have been described that give rise to loss-of-function and gain-of-function genetic disorders (Lacruz and Feske, 2015; Böhm and Laporte, 2018; Feske, 2010). Gain-of-function disorders primarily affect skeletal muscles and platelets, although other organs also seem to be affected. Given the rarity of the disorders, compared to other myopathies, their prevalence is currently unknown; they have not been tackled systematically in the clinic, and disease registries are not available at present. Both STIM1 and ORAI1 mutations are linked to three separate, but overlapping, disorders: tubular aggregate myopathy (TAM), Stormorken syndrome and York platelet syndrome. TAM is characterized by variable combinations of myalgias, cramps and muscle stiffness, with or without weakness with a predominantly proximal distribution (Böhm et al., 2014), and by the presence of tubular aggregates, which are regular arrays of tubules derived from the SR (Schiaffino, 2012). Stormorken syndrome (Stormorken et al., 1995) is variably characterized by myopathic signs, mild bleeding tendency due to platelet dysfunction, thrombocytopenia, anemia, asplenia, congenital miosis, ichthyosis, headache and recurrent stroke-like episodes (Nesin et al., 2014; Misceo et al., 2014; Noury et al., 2017). In York platelet syndrome, blood dyscrasias is the main phenotype (Markello et al., 2015). Fig. 1A illustrates the mutations reported so far. Briefly, mutations of STIM1 mostly reside in the EF-hand Ca^2+^-binding motifs, most likely modifying the affinity for Ca^2+^ ions of the protein, with few exceptions located on the cytosolic coiled-coil domains. The R304W mutation has recently been shown to affect the dimerization and resting state of STIM1 (Fahrner et al., 2018). A second substitution at the same position (R304Q; Harris et al., 2015) and a deletion on the third coiled-coil domain have also been reported (I484R fs*21; Okuma et al., 2016). The mutations of ORAI1 are located in the transmembrane domains in positions that might lead to the assumption that the resultant amino acids participate in the channel lining.

There are three reported mouse models bearing gain-of-function mutations of STIM1. The first model originated from a mutation of STIM1 (STIM1*^Sax^*; D84G), generated via random chemical mutagenesis, located in the intraluminal EF-hand domain. STIM1*^Sax^* is characterized by severe thrombocytopenia, but no muscular phenotype was reported (Grosse et al., 2007). Recently, two groups have described mice bearing the R304W mutation, which leads to Stormorken syndrome (Silva-Rojas et al., 2018; Gamage et al., 2018). Both groups found bleeding disorders and a muscular phenotype. Surprisingly, no models exist that investigate and reproduce clinically relevant mutations located on the luminal side of STIM1.

In this study, we have generated and characterized a knock-in animal bearing a mutation in one of the two EF-hand motifs. Heterozygous mice bearing the I115F mutation display important histological and functional muscle dysfunctions associated with thrombocytopenia, and unexpected hematological defects related to the myeloid lineage and natural killer (NK) cells.

## RESULTS

### Development of the KI*-*STIM1^I115F^ mouse colony

The knock-in (KI) STIM1^I115F^ mouse model was generated by homologous recombination by PolyGene Transgenetics. Details are provided in the Materials and Methods section.

Both male and female mice are fertile. Given that the disorder is a dominant gain-of-function, it was decided to routinely breed wild-type (WT) and heterozygous (KI*-*STIM1^I115F^) mice. The genotype ratio of born pups was 120:118 and did not significantly deviate from the expected Mendelian ratio of 1:1. Average litter size was 7.2+0.5 (*n*=36 litters). Anecdotally, two matings were attempted between heterozygous animals. One of the matings did not result in a litter, while the other resulted in two pups (one WT and one heterozygous). Given that characterization of homozygosity was not an aim, we did not analyze whether this was a result of embryonic lethality, and we did not breed between heterozygous animals further.

The Kaplan–Meier of survival shows that there are no signs of early mortality, and KI*-*STIM1^I115F^ mice survive over 1 year, like WT animals (Fig. 1B). Animals were not bred for longer, and therefore data are not sufficient to define the median survival of animals and whether the mutation leads to a decreased lifespan. With age, KI*-*STIM1^I115F^ mice may display an arched back that worsens with age, may limp and show tremors, and manifest an instantaneous post-mortem rigidity.

On visual inspection, KI*-*STIM1^I115F^ mice are smaller than WT mice, and this rendered blinding difficult for behavioral tests. The size difference (Fig. 1C) could be better appreciated when mice were weighed, and a significant difference between KI*-*STIM1^I115F^ and WT could be observed at all time points evaluated (1, 3, 6, 12 months), when net weight or body weight adjusted for length (anus-nose) were considered (Fig. 1D). Animal length was not significantly different between KI*-*STIM1^I115F^ and WT mice, also when normalized for tibial length.

At necropsy, the weights of the heart and spleen from KI*-*STIM1^I115F^ animals were determined and were not statistically different from those of WT (Fig. 1E,F). However, when body weight was taken into account, a statistically significant increase in heart size was evident at 12 months, while an increased spleen/body weight ratio was already observable at 3 months.

We also proceeded to analyze whether males (50 WT and 58 KI*-*STIM1^I115F^) and females (71 WT and 59 KI*-*STIM1^I115F^) differed. We did not find any significant differences between the sexes regarding survival at 1 year, body weight, animal length, heart size and spleen size (Figs S1 and S2), thereby suggesting that there is not a sex difference in penetrance of the disease.

### Myotubes from the KI*-*STIM1^I115F^ mouse colony retain increased SOCE

We next examined whether myotubes from KI*-*STIM1^I115F^ mice retained the fundamental cellular characteristic of increased Ca^2+^ entry upon store depletion (SOCE). Myotubes were generated from four animals in each condition. We also analyzed whether SOCE in WT myotubes was modified during the lifespan of these animals. To do this, we employed a classical protocol for Ca^2+^ entry, in which stores are depleted with 2,5-t-butylhydroquinone (tBHQ) in a Ca^2+^-free buffer, and, after 10 min, cells are perfused in a Ca^2+^-containing solution (2 mM).

**Fig. 2.**
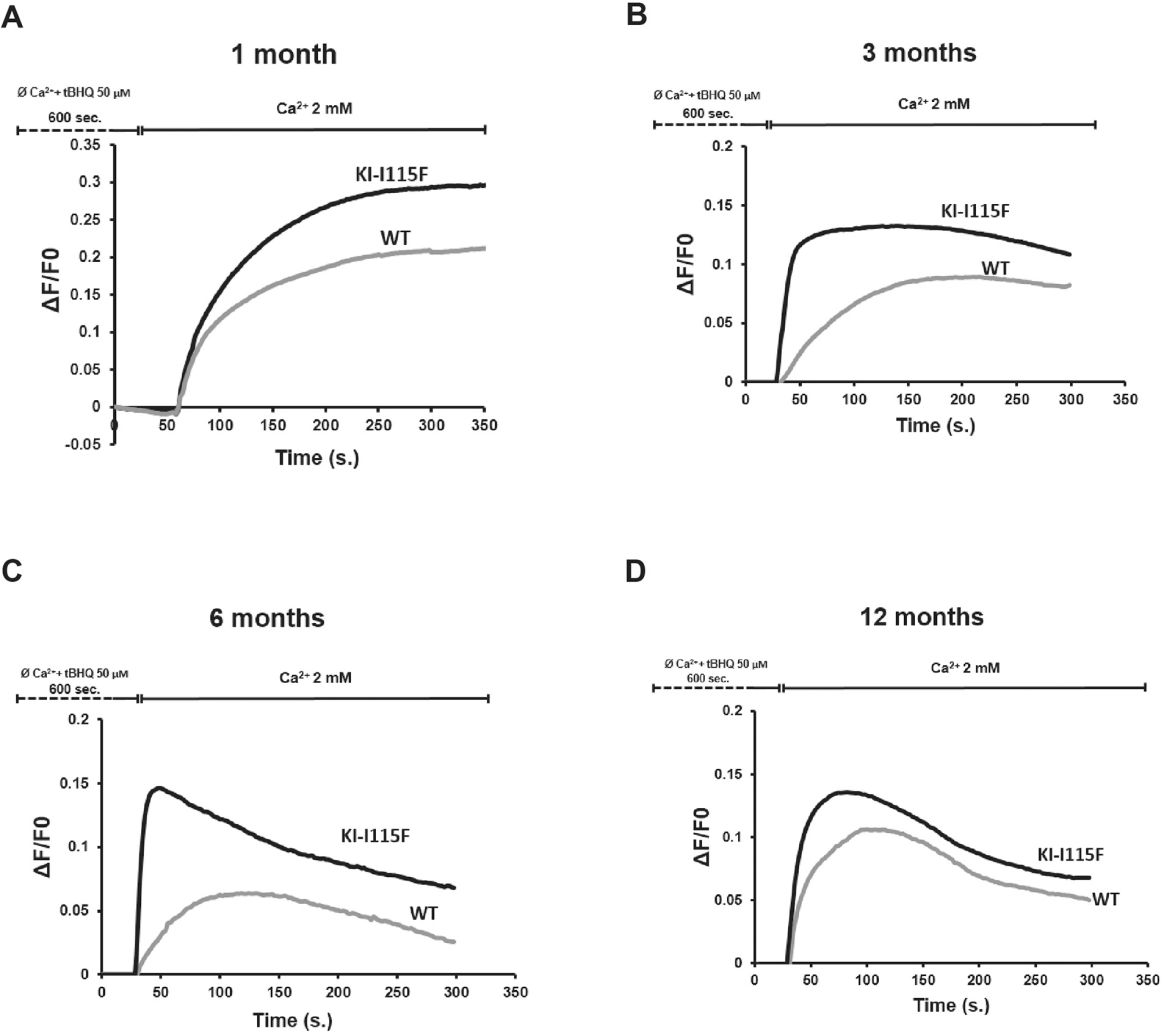
**SOCE alterations in myotubes.** Evaluation of SOCE by calcium imaging in myotubes from WT and KI-I115F at (A) 1, (B) 3, (C) 6 and (D) 12 months of age. Traces are the average of at least 180 myotubes from 6-well plates on two different experimental days. At all time points, myotubes from four animals (two males, two females) for each condition were used.

As can be observed in Fig. 2, myotubes from KI*-*STIM1^I115F^ display a significantly augmented SOCE compared to WT at 1 month. The increased SOCE was retained throughout all time points examined (1, 3, 6, and 12 months). Interestingly, SOCE from WT myotubes appeared to be higher in younger animals (1 month) and decreased at the subsequent time points. It is also interesting to note that, in myotubes from mice at 6 and 12 months, there was a reduced sustained entry compared to the earlier time points.

We next analyzed the Ca^2+^ entry pattern and found that slope, peak Ca^2+^ entry and area under the curve differed between KI*-*STIM1^I115F^ and WT mice, except at 1 month, when the slope appeared similar (Fig. 2; Fig. S3). We also investigated whether we could observe any spontaneous oscillations in intracellular Ca^2+^, or whether there were changes in basal Ca^2+^ between WT and KI*-*STIM1^I115F^ mice. No difference in basal Ca^2+^ between the two groups was observed when considering the same age group. Furthermore, analysis for 20 min of ∼50 cells per time point did not yield any oscillations, in any of the two strains.

We evaluated whether Ca^2+^ entry genes (*Trpc1*, *Trpc2*, *Trpc3*, *Trpc4*, *Trpc5*, *Orai1*, *Orai2*, *Orai3*, *Stim1* and *Stim2*) were modified in their expression between the two groups at 1, 3, 6, and 12 months (Fig. S4). No major differences were observed by quantitative real-time PCR (qRT-PCR). Indeed, we did not observe a compensatory decrease in *Stim1* mRNA expression. Although we did observe some minor, albeit statistically significant, differences at different time points, these were not consistent and are most likely a result of bias for multiple testings.

### Muscles in KI*-*STIM1^I115F^ mice present significant histological and functional alterations

**Fig. 3.**
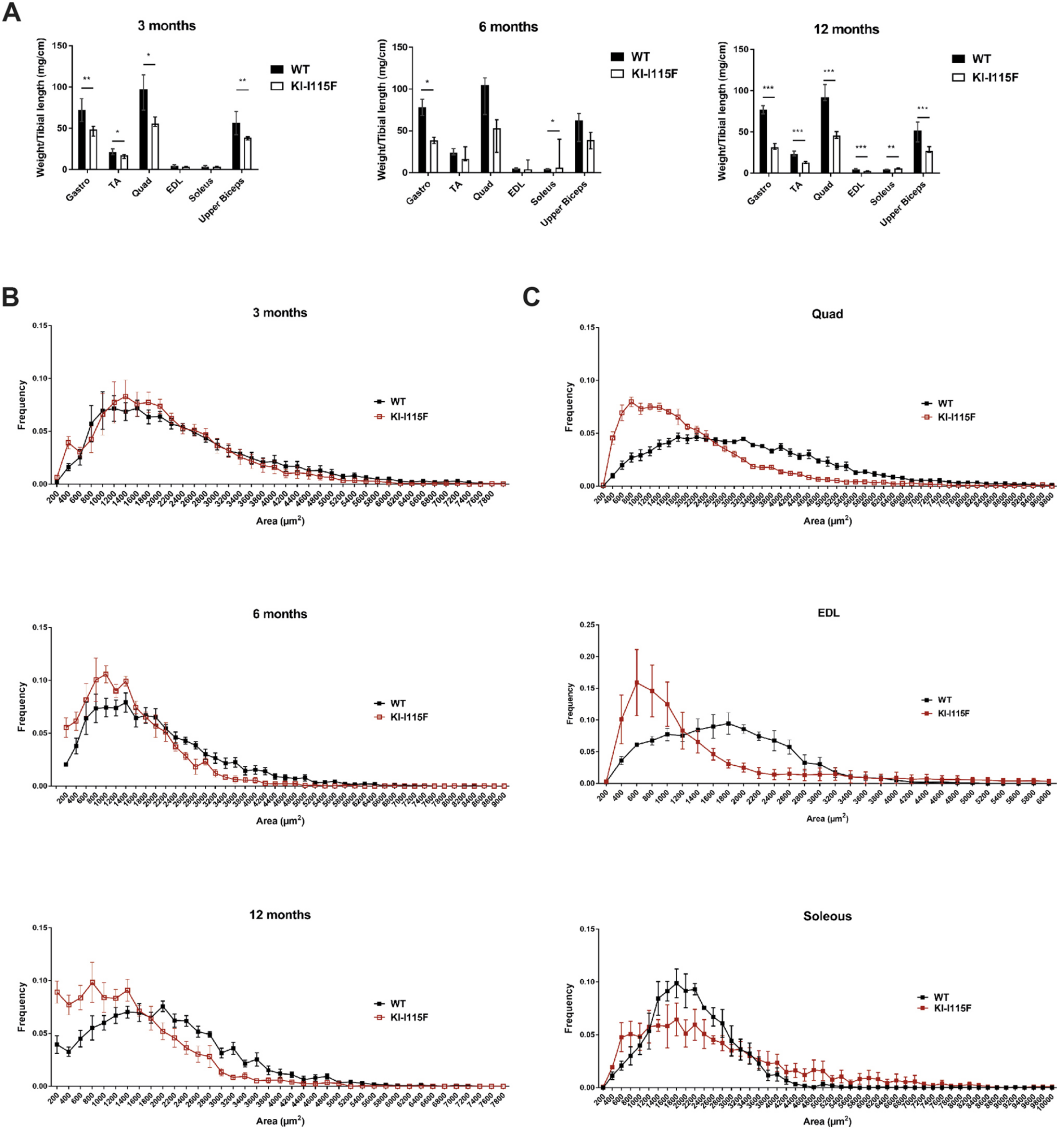
**Reduced muscle growth and damage in KI*-*STIM1^I115F^ mice.** (A) Muscle weight in WT and KI-I115F mice at 3 (WT *n*=6, KI-I115F *n*=6), 6 (WT *n*=4, KI-I115F *n*=4) and 12 (WT *n*=8, KI-I115F *n*=8) months. (Gastro, gastrocnemius; TA, tibialis anterior; Quad, quadriceps; EDL, extensor digitorum longus). Histograms show median and IQR of muscle weight. Mann–Whitney *U*-test. *P≤0.0303, **P≤0.0022, ***P≤0.0003 versus WT. (B) Cross-sectional area (CSA) frequency distribution of fibers in TA at 3 (WT *n*=6, KI-I115F *n*=6), 6 (WT *n*=4, KI-I115F *n*=4) and 12 (WT *n*=6, KI-I115F *n*=6) months. (C) CSA frequency distribution of fibers in quadriceps (Quad, top; WT *n*=4, KI-I115F *n*=3), extensor digitorum longus (EDL, middle; WT *n*=3, KI-I115F *n*=4) and soleus (bottom; WT *n*=3, KI-I115F *n*=3) at 6 months.

We then analyzed muscle weight in animals of different ages. As shown in Fig. 3A, the gastrocnemius and quadriceps muscles in KI*-*STIM1^I115F^ animals weigh significantly less compared to in WT animals at 3 months (no change was observed at 1 month; data not shown), and all muscles evaluated, except for the soleus, weigh less in KI*-*STIM1^I115F^ than in WT animals at 12 months. Similar trends were observed when a subanalysis was performed between male and female animals (Fig. S5). The paradoxical finding of the increased weight of the soleus at 6 and 12 months parallels the report on R304W mice by Silva-Rojas et al. (2018). Cross-sectional area (CSA) analysis of the tibialis anterior (TA) fibers shows a higher frequency of smaller areas in KI*-*STIM1^I115F^ animals compared to WT, and this worsens with age (Fig. 3B). A similar trend was observed in the quadriceps and extensor digitorum longus, but not in the soleus (Fig. 3C), in accord with the data obtained from muscle weight analyses (Fig. 3A).

**Fig. 4.**
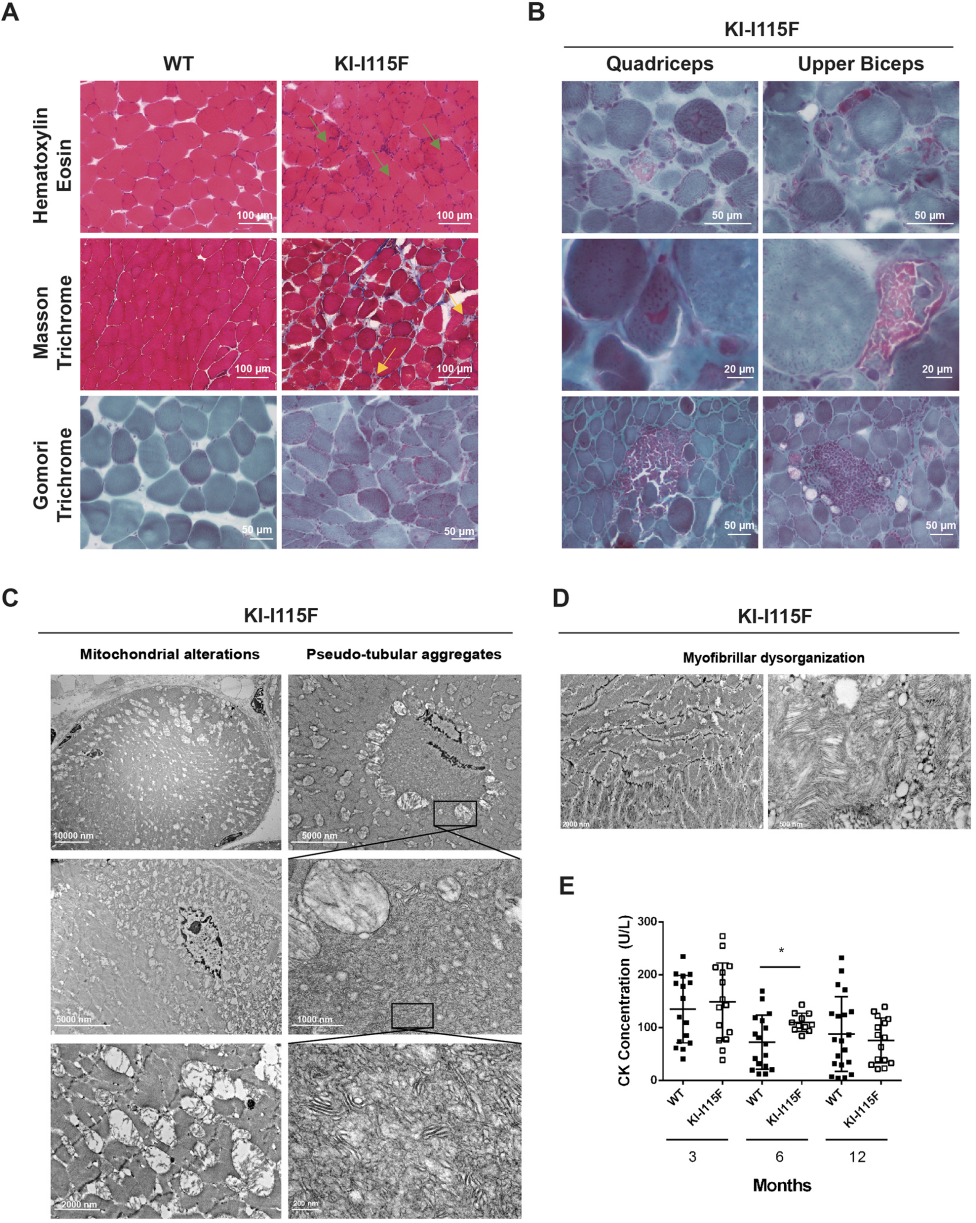
**Muscle histology of KI*-*STIM1^I115F^ mice.** (A) Representative images of Hematoxylin-Eosin (top row), Masson Trichrome (middle row) and Gomori Trichrome (bottom row) staining of the quadriceps at 6 months of age. Green arrows indicate fibrotic tissue and inflammatory cell infiltration; yellow arrows indicate necrotic fibers. (B) Representative images of Gomori Trichrome staining in the quadriceps (left column) and upper biceps (right column) from 6-month-old KI-I115F mice that show cytoplasmic fuscinophilic areas (top and middle rows) and inflammatory infiltrates (bottom row). (C) Representative electron microscopic images of the quadriceps of 6-month-old KI-I115F mice, showing pseudo-tubular aggregates (right column) and mitochondrial alterations (left column). (D) Representative electron microscopic images of the quadriceps of 6-month-old KI-I115F mice, showing myofibrillar dysorganization. (E) Creatine kinase (CK) plasma concentrations. Mean±s.e.m. at 3 (WT *n*=16, KI-I115F *n*=15), 6 (WT *n*=17, KI-I115F *n*=11) and 12 (WT *n*= 20, KI-I115F *n*=15) months. *P=0.011 versus WT.

Histological sections of the quadriceps and upper biceps were obtained to evaluate muscle morphology. Hematoxylin-Eosin, Masson Trichrome and Gomori Trichrome stainings were performed (Fig. 4A,B). A myopathic process was evident from 1 month and worsened with age. It was characterized by marked fiber size variability, necrosis and regeneration with newly formed myofibers with central nuclei, and increased connective tissue. Interestingly, in the quadriceps and upper biceps of 6-month-old animals, endomysial inflammatory infiltrates as well as cytoplasmic fuscinophilic areas in Gomori Trichrome staining were observed (Fig. 4B). In electron microscopy of 6-month-old animals (quadriceps and upper biceps), enlarged mitochondria with abnormal morphology were observed (for example, hypertrophic with loss of the mitochondrial crest; Fig. 4C, left column), which at times formed circular structures (Fig. 4C, top left). Some mitochondria were located in a subplasmallemal position (Fig. 4C, middle and bottom left), which might explain some features observed in Gomori staining (e.g. Fig. 4B, bottom row). Inside the circular structures formed by mitochondria, no sarcomeric structures were observed, although pseudo-aggregate structures were at times visible (Fig. 4C, middle and bottom right), and they resembled, in part, the characteristics of the human disorders, but were disorganized and chaotic. However, no true tubular aggregates were observed at either 6 or 12 months, and these pseudo-aggregates were not observable in 12-month-old animals. These features were accompanied by a partial disorganization of myofibrils (Fig. 4D). Subsequently, we proceeded to analyze myosin in fibers. The frequency distribution of type IIa fibers was increased in the quadriceps, whereas no differences were observed in the extensor digitorum longus. In the soleus, both type IIa and type I fibers were decreased in KI*-*STIM1^I115F^ compared to WT animals, but this was paralleled by a marked increase in fibers that were negative for both type IIa and type I myosin (Fig. S6).

Despite the marked histopathological alterations in muscle tissue, only small differences in serological creatine kinase (CK) levels were observed between KI*-*STIM1^I115F^ and WT during the entire lifespan (Fig. 4E). A significant variability in CK levels was observed, presumably due to the low levels detected. Similar trends were observable in a subanalysis of male and female animals (Fig. S7).

**Fig. 5.**
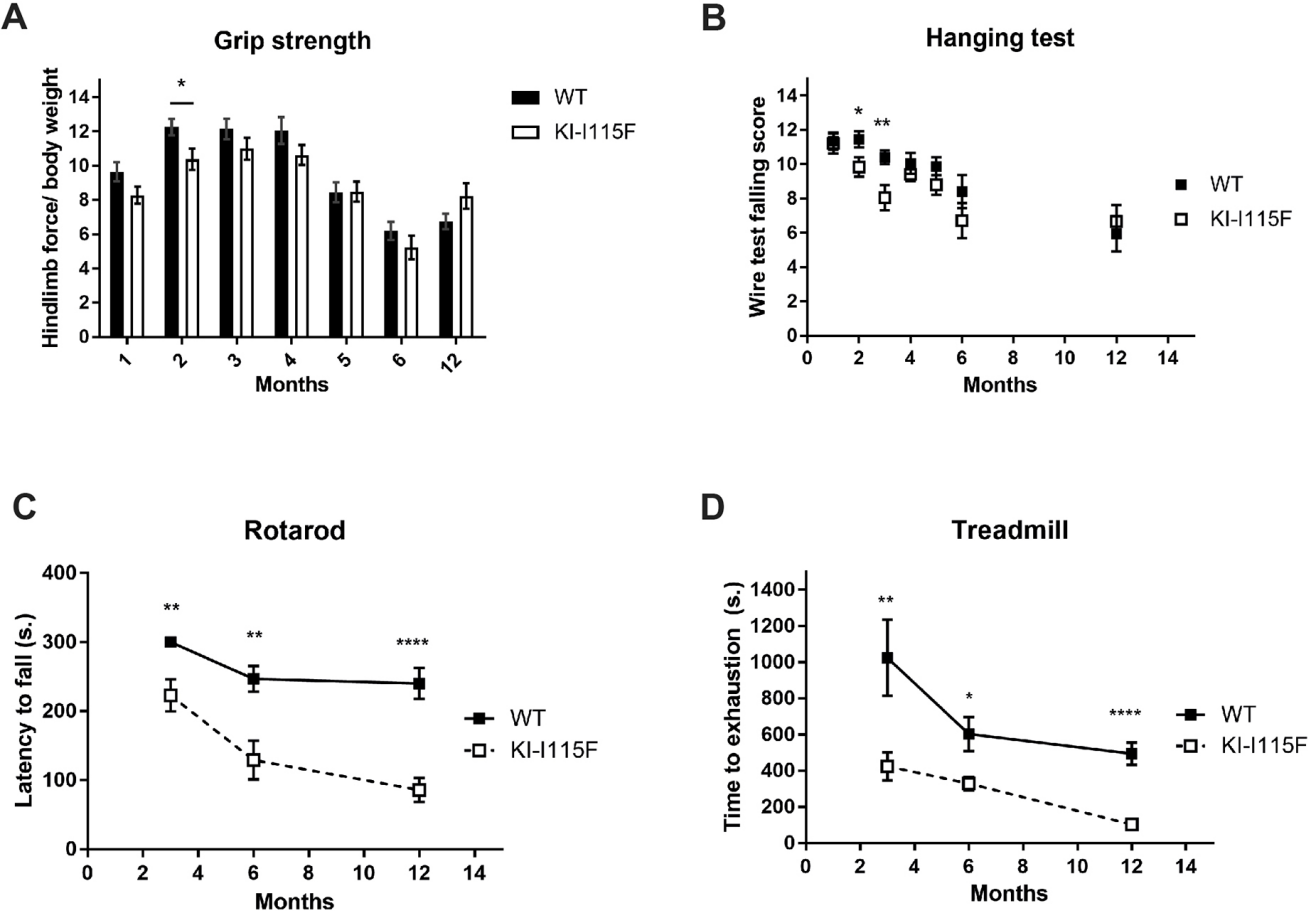
**Characterization of muscular functionality of KI*-*STIM1^I115F^ mice.** (A) Grip strength performance at 1 (WT *n*=45, KI-I115F *n*=40), 2 (WT *n*=35, KI-I115F *n*=27), 3 (WT *n*=39, KI-I115F *n*=34), 4 (WT *n*=34, KI-I115F *n*=30), 5 (WT *n*=34, KI-I115F *n*=25), 6 (WT *n*=19, KI-I115F *n*=14) and 12 (WT *n*=13, KI-I115F *n*=12) months. Graph shows the means±s.e.m. of the falling score. Unpaired Student's *t*-test with Welch's correction. *P=0.02 versus WT. (B) Hanging test performance at 1 (WT *n*=45, KI-I115F *n*=37), 2 (WT *n*=37, KI-I115F *n*=32), 3 (WT *n*=42, KI-I115F *n*=33), 4 (WT *n*=35, KI-I115F *n*=29), 5 (WT *n*=32, KI-I115F *n*=26), 6 (WT *n*=17, KI-I115F *n*=14) and 12 (WT *n*=18, KI-I115F *n*=18) months. Graph shows the means±s.e.m. of the score (see Materials and Methods). Unpaired Student's *t*-test with Welch's correction. *P=0.0312, **P=0.0064 versus WT. (C) Rotarod test performance at 3 (WT *n*=11, KI-I115F *n*=17), 6 (WT *n*=13, KI-I115F *n*=10) and 12 (WT *n*=13, KI-I115F *n*=13) months. The 3-month data were analyzed by one-sample Student's *t*-test KI-I115F versus 300 s. All WT mice achieved the endpoint of the experiment (300 s). Graph shows the means±s.e.m. of the latency to fall in WT and KI-I115F. Unpaired Student's *t*-test with Welch's correction. **P=0.0034 KI-I115F versus 300 s. **P=0.0096, ****P<10^−4^ versus WT. (D) Treadmill test performance at 3 (WT *n*=7, KI-I115F *n*=9), 6 (WT *n*=15, KI-I115F *n*=15) and 12 (WT *n*=16, KI-I115F *n*=8) months. Graph shows the means±s.e.m. of the time to exhaustion. Unpaired Student's *t*-test with Welch's correction. *P=0.0116, **P=0.0041, ****P<10^−4^ versus WT.

Finally, we analyzed the motor and strength performances of the animals. KI*-*STIM1^I115F^ mice performed similarly to WT mice, both in the grip strength and hanging test, although a reduced performance was evident at around 2-3 months, which then recovered to levels comparable to WT (Fig. 5A,B). In contrast, when animals were tested on the rotarod or the treadmill, an underperformance of KI- STIM1^I115F^ with respect to WT was already evident at 3 months (the earliest age at which these tests can be performed reproducibly) and was maintained up to 1 year (Fig. 5C,D).

### KI*-*STIM1^I115F^ mice display hematological defects

**Fig. 6.**
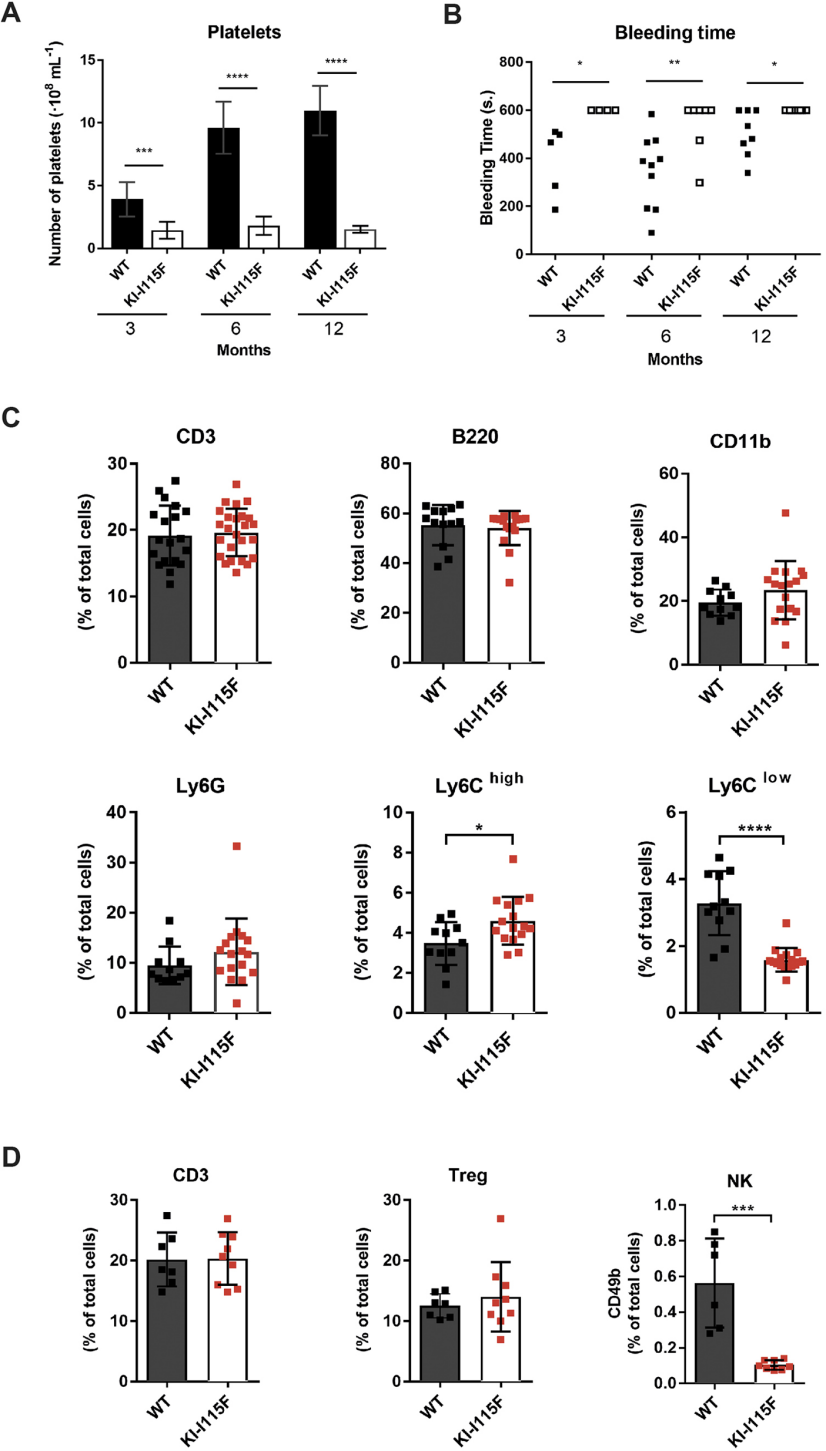
**Evaluation of hematological defects in KI*-*STIM1^I115F^ mice.** (A) Platelet count at 3 (WT *n*=8, KI-I115F *n*=7), 6 (WT *n*=9, KI-I115F *n*=11) and 12 (WT *n*=8, KI-I115F *n*=10) months. Histogram shows the means±s.e.m. of the number of platelets in WT and KI-I115F. Unpaired Student's *t*-test with Welch's correction. ***P=0.0015, ****P<10^−4^ versus WT. (B) Bleeding time at 3 (WT *n*=5, KI-I115F *n*=4), 6 (WT *n*=10, KI-I115F *n*=7) and 12 (WT *n*=8, KI-I115F *n*=8) months. Scatter plots show bleeding time. Unpaired Student's *t*-test with Welch's correction. *P≤0.0256, **P=0.0064 versus WT. (C) CD3 (CD45^+^CD3^+^), B220 (CD45^+^B220^+^), CD11b (CD45^+^CD11b^+^), Ly6G (CD45^+^CD11b^+^Ly6G^+^), Ly6C^high^ (CD45^+^Cd11b^+^Ly6G^−^Ly6C^high^) and Ly6C^low^ (CD45^+^CD11b^+^Ly6G^−^Ly6C^low^) cell populations in blood from mice at 6 months (WT *n*=11, KI-I115F *n*=17). Unpaired two-tailed Student's *t*-test was used for statistical analysis. *P=0.0193, ****P<0.0001 versus WT. (D) CD3 (CD45^+^CD3^+^), Treg (CD4^+^Foxp3^+^CD25^+^) and NK (CD3^−^CD11b^−^CD49b^+^) cells in spleen from mice at 6 months (WT *n*=6, KI-I115F *n*=8). Unpaired two-tailed Student's *t*-test was used for statistical analysis. ***P=0.0002 versus WT.

We next proceeded to determine platelet count in animals at 3, 6 and 12 months. As shown in Fig. 6A, significant thrombocytopenia was evident in KI*-*STIM1^I115F^ mice of all ages. This reduced number of platelets was reconciled with an increased bleeding time, which was evaluated via the tail test (Fig. 6B). Of the 19 KI*-*STIM1^I115F^ mice tested, only two stopped bleeding within 10 min of the incision, compared to 20 out of 23 in the WT animals.

To gain an insight into other hematological dyscrasias, we next evaluated the frequency of circulating B and T lymphocytes in WT or KI*-*STIM1^I115F^ mice at 6 months of age. As shown in Fig. 6C (top row), we could not detect any difference in the frequency of T cells (CD45^+^CD3^+^cells), B cells (CD45^+^B220^+^cells) and CD11b^+^ cells (CD45^+^Cd11b^+^) between KI*-*STIM1^I115F^ and WT. We also evaluated the myeloid populations, and found that KI*-*STIM1^I115F^ mice displayed similar levels of total granulocytes (CD45^+^Cd11b^+^Ly6G^+^) compared to WT (Fig. 6C, bottom row). In contrast, KI*-*STIM1^I115F^ showed impairment of the monocyte subsets. As shown in Fig. 6C (bottom row), the number of Ly6C^high^ monocytes (CD45^+^Cd11b^+^Ly6G^−^Ly6C^high^ cells) was increased in KI*-*STIM1^I115F^ mice, with a marked reduction in the levels of Ly6C^low^ monocytes (CD45^+^Cd11b^+^Ly6G^−^Ly6C^low^cells), compared to WT. These data demonstrate that KI*-*STIM1^I115F^ mice have a normal T and B subset, but suggest an alteration in monocyte differentiation. Furthermore, to investigate the impact of the mutation on the immune system, we quantified regulatory T (Treg) (CD4^+^FOXp3^+^CD25^+^) and NK (CD3^−^CD11b^−^CD49b^+^) cells in the spleen of WT and KI*-*STIM1^I115F^ mice. As shown in Fig. 6D, only the number of NK cells was significantly reduced in KI*-*STIM1^I115F^ compared to WT animals, suggesting a putative role of the STIM1 p.I115F mutation in the differentiation/maturation of NK cells (Abel et al., 2018). The gating strategies of fluorescence-activated cell sorting (FACS) analyses are reported in Fig. S8.

## DISCUSSION

TAM, Stormorken syndrome and York platelet syndrome represent a cluster of ultra-rare genetic diseases that can be attributed to overactivation of SOCE, a fundamental mechanism that allows calcium replenishment after ER store emptying. These disorders are driven by mutations in one of two key proteins involved in SOCE (STIM and ORAI). These mutations lead to gain-of-function proteins, and the clinical hallmarks are muscle weakness and platelet dysfunction. The two hallmarks may have variable penetrance in each individual, also according to the mutation.

In the present study, we generated a mouse colony bearing the I115F (c.343A>T) constitutive knock-in point mutation (KI*-*STIM1^I115F^), on a C57Bl/6 background. This mutation is located in one of the two luminal EF-hand Ca^2+^-binding motifs, and has been associated in the clinic with TAM and York platelet syndrome (Lacruz and Feske, 2015). Although a mouse bearing a mutation in the EF-hand motifs (STIM1*^Sax^*; D84G) has been described, the muscle phenotype was either not present or was not investigated (Grosse et al., 2007), as only a severe thrombocytopenia was reported. Two independent groups have reported the generation and characterization of mouse colonies that bear the R304W mutation, which is characteristic of TAM and Stormorken syndrome (Silva-Rojas et al., 2018; Gamage et al., 2018). The R304W mutation is located on the cytosolic domains of STIM1, and therefore our model is perfectly complementary to that described earlier.

KI*-*STIM1^I115F^ mice are fertile, breed well in heterozygosity and ∼50% of newborns display the mutation. The Mendelian ratio obtained when heterozygous animals were mated with WT was 1:1, which was as expected. This is partly in contrast to results reported by Silva-Rojas et al. (2018) for STIM1^R304W^, as fewer than expected heterozygous pups were observed.

KI*-*STIM1^I115F^ mice are lighter than WT mice, which might reconcile with the decreased muscle weight that is observed. Importantly, this manifestation can be observed at 1 month of age, making it a good surrogate early endpoint to investigate drugs in the future. Similarly, platelet dysfunction can also be observed early, providing a further early read-out.

KI*-*STIM1^I115F^ mice showed a normal performance in the grip strength and hanging tests. This somehow differs from the model described by Silva-Rojas et al. (2018), in which STIM1^R304W^ mice showed impaired performance at 9 weeks (the only time assessed). We found, instead, a strong impairment in both the rotarod and treadmill tests, which also contrasts with observations by Silva-Rojas et al. (2018) (at 9 weeks) and Gamage et al. (2018) (at 5-6 months). Our model, therefore, appears to show functional deficits in tests that evaluate motor coordination (rotarod test) and speed activity (treadmill test), suggesting a prevalent impairment in actions that require running or sprinting, a deficit also reported in TAM patients (Walter et al., 2015). The negative performance of KI*-*STIM1^I115F^ mice in the rotarod test is unlikely to be attributable to balance impairment, as this is not supported by the hanging test (a functional test that also requires coordination), demonstrating muscle involvement but not a cerebellar deficit.

In line with the Ca^2+^ overload detected in myotubes from TAM patients, in KI*-*STIM1^I115F^ myotubes, we observed a SOCE overactivation that was maintained during the entire lifespan, unlike what was observed in WT mice. However, in contrast to previous data reported in TAM patient myotubes (Garibaldi et al., 2016), we did not observe any increase in basal calcium levels or spontaneous oscillations in KI*-*STIM1^I115F^ myotubes. This observation at present has no explanation, although preliminary evidence reveals that platelets from KI*-*STIM1^I115F^ animals instead show an increased basal Ca^2+^ level (C.C.-S., unpublished).

Histopathological findings in KI*-*STIM1^I115F^ mice revealed a progressive muscle degeneration. Muscle tissue showed histological features consistent with a myopathic process, similar to those observed in the other mice models (Silva-Rojas et al., 2018; Gamage et al., 2018). Given the different times at which muscles were evaluated in the different models, comparison of the severity of the myopathy is not possible. In our model, at 12 months, muscles showed a severe myopathic process. When evaluating different muscles at 6 months, we found mitochondrial alterations and the presence of aggregates that, in part, may represent the counterpart of the human hallmark, although they were not superimposable. It is worth noting, therefore, that all three models lack true tubular aggregates (Silva-Rojas et al., 2018; Gamage et al., 2018), although the pseudo-aggregates observed in KI*-*STIM1^I115F^ mice will require more investigation in the future. Interestingly, we detected in some mice abundant endomysial inflammatory infiltrates, a pathological feature that has been reported in a mouse model overexpressing STIM1 (Goonasekera et al., 2014). In KI*-*STIM1^I115F^, the myopathic process was not paralleled by significant increases in CK levels. Although an increasing trend was observed, the variability, which might have been due to the technology used or to true inter-individual variability, did not lead to consistent statistical significance.

As reported in patients carrying the STIM1 p.I115F mutation, our mouse model also displays a severe platelet dysfunction. At all time points evaluated, we observed a marked reduction in platelet number, which is likely to be linked to the increased bleeding time. It has previously been reported in the STIM1*^Sax^* mouse that thrombocytopenia is associated with increased basal calcium in platelets, resulting in a pre-activation state (Grosse et al., 2007). Such a mechanism would be compatible with our findings; indeed, in preliminary experiments, we confirmed this increase in basal Ca^2+^. Importantly, the two R304W models have contradictory findings on platelets: whereas Silva-Rojas et al. (2018) found a significantly decreased platelet number, Gamage et al. (2018) found no differences in heterozygous animals and attributed this negative finding to a compensatory reduction in STIM1 protein. The KI*-*STIM1^I115F^ model did not display compensatory mechanisms, at least at the mRNA level.

Considering the crucial role of SOCE in the immune system (Feske et al., 2010) and the involvement of STIM and ORAI loss-of-function mutations in severe immunodeficiencies (Lacruz and Feske, 2015), we investigated the impact of the STIM^I115F^ mutation on circulating T lymphocytes, B lymphocytes, neutrophils and monocytes. In contrast to observations by Silva-Rojas et al. (2018), in STIM^R304W^, we did not find impairment of T cells or a significant increase in the levels of neutrophils, but found alterations in monocyte subsets and in NK cells, which are worth exploring in patients in the future.

To date, 12 point mutations have been reported on the intraluminal EF-hand motifs and four mutations/deletions described in the cytosolic coiled-coil domains of STIM1. Given the extremely low frequency of these mutations, it is plausible that only drugs that will be able to counteract most of these mutations will find sufficient patients to treat in clinical trials and be approved. In this respect, we believe the presence of complementary models mimicking luminal and cytosolic mutations will increase the likelihood of success for the development of effective drugs for patients that are – at present – lacking therapeutic options.

## MATERIALS AND METHODS

### KI-STIM1^I115F^ mouse model generation and animal care

The care and husbandry of animals were in conformity with the institutional guidelines, in compliance with national and international laws and policies. Mice were housed in ventilated cages in 22±1°C monitored rooms with 12 h light/dark cycles, had access to food and water *ad libitum*, and were weaned at 23 days by sex. The procedures were approved by the local animal-health and ethical committee (Università del Piemonte Orientale) and authorized by the national authority (Istituto Superiore di Sanità; authorization number N. 194/2019-PR).

KI*-*STIM1^I115F^ founders in a C57Bl/6N background were obtained from PolyGene Transgenics (https://www.polygene.ch/). Briefly, this knock-in mouse model was generated by homologous recombination in electroporation-transfected embryonic stem (ES) cells on exon 3 of the *Stim1* gene, located on chromosome 7, inserting the c.343A>T mutation (corresponding to isoleucine to phenylalanine substitution; I115F). The linearized targeting vector F118.3 TV, with a flippase recognition target (FRT)-flanked neomycin resistance cassette, inserted in an unsuspicious region in intron 3 of *Stim1*, was used for the electroporation. The integrity of the targeting vector was confirmed by sequencing exonic regions and by restriction analysis. G418 selection was used to maintain stable transfection, and the clones obtained were analyzed and validated by PCR and Southern blotting using BstEII-digested DNA (internal probe) and a 3′ external probe (LA probe).

The selected ES clones were injected into 49 blastocysts from gray C57Bl/6N mice. Forty-one surviving blastocysts were transferred to two CD-1 foster mice. Resulting chimeras were mated to gray Flp-deleter mice. The offspring from the chimeras were screened for the Flp-mediated deletion of the neomycin cassette and the corresponding presence of the remaining FRT site.

Mice were identified with the ear punch method at weaning and the piece of tissue obtained was used to perform genotyping using a PCRBIO Rapid Extract PCR Kit (PCR Biosystems, UK). DNA extraction was performed according to the manufacturer's instructions (5× PCRBIO Rapid Extract Buffer A, 10× PCRBIO Rapid Extract Buffer B). For tissue lysis, the samples were incubated at 75°C for 5 min. Another incubation at 95°C for 10 min was performed to deactivate proteases. Subsequently, the reaction was stopped by adding mQ H_2_O, and centrifugation at 13,000 ***g*** for 1 min was performed to eliminate the debris. The PCR reaction was conducted using the following primers: FW, 5′-CCAGCAACTGAGGCATTC-3′; REV, 5′-AAGAGGTGGAGTAGGCAGAG-3′. A single band was obtained as a final PCR product in WT mice (617 bp), whereas a double band was obtained in heterozygotes KI*-*STIM1^I115F^ (617 bp and 746 bp), related to the presence of the remaining FRT site.

### Bleeding time

From an isoflurane-anesthetized animal, a segment of 2 mm was cut from the tail tip and immersed in a solution of 0.9% NaCl (Sigma-Aldrich, Italy) at 37°C. Bleeding time was measured for a maximum of 10 min to avoid unnecessary suffering.

### Behavioral tests

All behavioral tests were performed according to the Standard Operating Protocols (SOPs) of the TREAT-NMD neuromuscular network (http://www.treat-nmd.eu/research/preclinical/dmd-sops/).

#### Grip strength test

To measure the forelimb force *in vivo*, mice were suspended by the tail and allowed to grasp on a rod. A force transducer, attached to the rod by the mice grasp, was used to measure the force and the time of peak resistance. Five trials per mouse were performed and data were normalized to mouse weight (DMD_M.2.2.001). Experiments were performed in WT and KI*-*STIM1^I115F^ mice at 1, 2, 3, 4, 5, 6 and 12 months.

#### Hanging wire test

To determine ‘subacute’ muscle function and coordination, mice were allowed to grasp onto the middle of a wire. Mice attempted to reach one of the wire ends and the number of ‘falls’ or ‘reaches’ was recorded. From an initial score of 10, each fall decreased the score by one and each reach increased the score by 1. The experiment was terminated after 180 s or when the animal reached zero as a score, whichever was earlier. A Kaplan–Meier-like curve was created with all data obtained (DMD_M.2.1.004). Experiments were performed in WT and KI*-*STIM1^I115F^ mice at 1, 2, 3, 4, 5, 6 and 12 months.

#### Rotarod test

To assess motor coordination and balance, animals were placed on a rotarod with fixed speed (2.8 m/min or 10 rpm). Experiments were terminated after five falls or after 5 min, whichever was earlier. Before the test, mice were left to acclimatize to the experimental room, and mice habituation to the rotarod for 1 min at low speed was performed (ESLIM_010_001; Shiotsuki et al., 2010).

#### Treadmill test

To assess the resistance and fatigue of mice, enforced running on a treadmill with no inclination at a fixed speed (12 m/min) was performed. The experiment was terminated after 30 min or after the fifth grid-stop, whichever came earlier. An acclimation period of 15 min in the experimental room was needed, and training for a maximum of 30 min was performed the previous day. Following the SOPs, no electric shock was used on the grid and it was substituted by a gentle touch of the tail, requiring two experimenters for continuous supervision of this test (DMD_M.2.1.003, DMD_M.2.1.001).

### CK assay

Blood was collected from isoflurane-anesthetized mice by submandibular vein puncture. Approximately 250 µl of blood was collected into heparin tubes to obtain plasma by centrifuging samples at 1200 ***g*** for 10 min at room temperature (RT). CK determination was performed according to the manufacturer's instructions using a standard spectrophotometric method with enzyme-coupled assay reagent from Point Scientific (C7522-150). Absorbance at 340 nm was measured every minute for 2 min at 37°C to calculate enzyme activity. Duplicate measurements were performed on each serum sample.

### Histological analysis

Muscles were trimmed of tendons and adhering non-muscle tissue, mounted in Killik embedding medium (Bio-optica, Italy), frozen in liquid nitrogen-cooled isopentane and stored at −80°C.

For conventional histological techniques, 8- to 10-μm-thick cryostat sections were stained with Hematoxylin-Eosin, Masson Trichrome and modified Gomori Trichrome (GT), to reveal general muscle architecture and myopathological features.

For ultrastructural studies, small muscle specimens were fixed with glutaraldehyde (2%, pH 7.4), then fixed with osmium tetroxide (2%), dehydrated and embedded in resin. Longitudinally oriented ultra-thin sections were obtained at different depths from one to three small blocks, and stained with uranyl acetate and lead citrate. Ultra-thin sections of transversally oriented blocks were obtained for only the most significant findings. The grids were observed using a Morgagni Fei electron microscope (The Netherlands) and were photo-documented using a Mega View II Camera (SYS Technologies).

For CSA distribution, muscle slices were fixed in 4% paraformaldehyde (PFA), permeabilized with 0.2% Triton X-100 in 1% bovine serum albumin (BSA) for 15 min and blocked with 4% BSA for 30 min. They were then incubated for 1 h with anti-laminin antibody (1:200; Dako, Agilent Technologies) and for a further 45 min with secondary antibody (1:400; anti-rabbit Alexa Fluor 488, Thermo Fisher Scientific) at RT. Images were acquired using a Leica CTR5500 B fluorescent microscope (Leica Biosystems, Germany) with the Leica Application SuiteX 1.5 software. CSA of the total muscle fibers was quantified with ImageJ software (v1.49o). For myosin heavy chain studies, muscle slices were not fixed with PFA and were stained with anti-laminin antibody (1:200; Dako, Agilent Technologies), BA-D5, Myosin Heavy Chain Type I (IgG2b; 1:50), SC-71, Myosin Heavy Chain Type IIA (IgG1; 1:500), BF-F3 and Myosin Heavy Chain Type IIB (IgM; 1:5), all obtained from the Developmental Studies Hybridoma Bank (Iowa City, IA, USA).

### Characterization of blood cell population

#### T cell, B cell, Treg cell, NK cell, monocyte and granulocyte determination

Blood cells were collected from the eye vein of anesthetized mice, and splenocytes from the spleen after filtration through cell strainers (70 µm). Ammonium-chloride-potassium lysing buffer was used for the lysis of red blood cells. Blood cells were labeled for 15 min at 4°C with fluorochrome-conjugated monoclonal antibodies: CD45-FITC BD Biosciences Clone 30-F11 (1:100), Ly6G BV421 BioLegend Clone 1A8 (1:500), Ly6C BV605 BD Biosciences Clone AL-21 (1:500), CD3e BV786 BD Biosciences Clone 5OOA2 (1:500), CD45R (B220) PE BD Biosciences Clone RA3-6B2 (1:500) and CD11b PE-Cy7 eBioscience Clone M1/70 (1:500). Splenocytes were labeled for 15 min at 4°C with fluorochrome-conjugated monoclonal antibodies: CD3 BV786 BD Biosciences (1:500), CD11b BV510 BD Biosciences (1:500), CD49b PE eBioscience (1:500), CD4 BV650 BD Biosciences (1:500), CD25 FITC BioLegend (1:500) and Foxp3 PerCP-Cy5.5 eBioscience (1:100). Samples were acquired with a BD LSRII Fortessa (BD Biosciences) and analyzed with FlowJo software.

#### Number of platelets

To determine platelet number, 18 µl of blood extracted from the submandibular vein was mixed with 2 µl acid citrate dextrose (Sigma-Aldrich). Ten microliters of the mixture was then blended with 190 µl NaNH_3_ (Sigma-Aldrich) for 5 min at RT to induce the lysis of red blood cells. Then, 10 µl of this solution was diluted in 90 µl of phosphate-buffered saline (PBS). Counts were performed in a Burker chamber on a microscope with a 40× objective (Dhanjal et al., 2007).

### Cell cultures

WT and KI*-*STIM1^I115F^ primary myoblasts were obtained from the following muscles: gastrocnemius, TA, quadriceps femoris, extensor digitorum longus, soleus, biceps brachii and diaphragm. Each muscle was placed in a 60-mm dish in PBS, removed from the tendon, separated longitudinally and subsequently chopped into smaller pieces. The small fragments were incubated with Pronase^®^ (Protease, *Streptomyces griseus*, Calbiochem^®^, 25 KU) for 1 h at 37°C with shaking and neutralized with Dulbecco's modified Eagle medium (DMEM, Sigma-Aldrich), supplemented with 10% heat-inactivated fetal calf serum (Gibco, Italy), 50 mg/ml L-glutamine (Sigma-Aldrich), 10 U/ml penicillin and 100 mg/ml streptomycin (Sigma-Aldrich), and 1% chicken embryo extract (Sigma-Aldrich). Tissues were then chopped into smaller pieces and passed through pipettes of 10 ml and 5 ml, and the supernatant obtained was filtered in a 40-µm strainer and centrifuged at RT for 10 min at 1200 rpm (235 ***g***). The pellets were re-suspended and myoblasts were plated in a 100-mm dish in DMEM, supplemented with 10% heat-inactivated fetal bovine serum (FBS) (Gibco), 50 mg/ml L-glutamine (Sigma-Aldrich), 10 U/ml penicillin and 100 mg/ml streptomycin (Sigma-Aldrich), and 1% chicken embryo extract (Sigma-Aldrich) for 90 min at 37°C, in a 5% CO_2_ humidified atmosphere, to separate cells from debris. Supernatants were then centrifuged and plated in a 2% gelatine-treated 35-mm dish in DMEM (Sigma-Aldrich), supplemented with 20% heat-inactivated FBS (Gibco), 10% horse serum (Gibco), 50 mg/ml L-glutamine (Sigma-Aldrich), 10 U/ml penicillin and 100 mg/ml streptomycin (Sigma-Aldrich), 1% chicken embryo extract (Sigma-Aldrich) and 10 ng/ml fibroblast growth factor (Peprotech, UK) at 37°C, in a 5% CO_2_ humidified atmosphere for 6-7 days, with a medium change every 24-36 h.

For differentiation into myotubes, myoblasts were transferred for 48 h into differentiation medium consisting of DMEM with 5% horse serum and 1% penicillin-streptomycin.

For calcium-imaging experiments, myotubes were further maintained in the same medium for 24 h upon plating onto glass coverslips at a concentration of 20×10^4^ per well (24-mm diameter coverslips in 6-well plates) and maintained in DMEM supplemented with 10% heat-inactivated FBS (Gibco), 50 mg/ml L-glutamine (Sigma-Aldrich), and 10 U/ml penicillin and 100 mg/ml streptomycin (Sigma-Aldrich), at 37°C in a 5% CO_2_ humidified atmosphere. Experiments were performed 6-7 days after the extraction at cell passage number (P)2 and P3.

### Fura-2 Ca^2+^ experiments

Myotubes from WT and KI*-*STIM1^I115F^ mice were loaded with 5 µM Fura-2 AM in the presence of 0.02% Pluronic-127 (both from Life Technologies, Italy) and 10 µM sulfinpyrazone (Sigma-Aldrich) in Krebs–Ringer buffer (KRB; 135 mM NaCl, 5 mM KCl, 0.4 mM KH_2_PO_4_, 1 mM MgSO_4_, 5.5 mM glucose, 20 mM HEPES, pH 7.4) containing 2 mM CaCl_2_ (30 min, RT). Then, cells were washed and incubated with KRB for 30 min to allow the de-esterification of Fura-2.

To measure SOCE, changes in cytosolic Ca^2+^ were monitored upon depletion of the intracellular Ca^2+^ stores. Experiments were carried out prior to and during exposure of the cells to the Ca^2+^-free solution. In the absence of Ca^2+^, the intracellular Ca^2+^ stores were depleted by inhibition of the vesicular Ca^2+^ pump by tBHQ (50 µM; Sigma-Aldrich). Re-addition of 2 mM Ca^2+^ allowed assessment of the SOCE.

During the experiments, the coverslips were mounted into an acquisition chamber and placed on the stage of a Leica DMI6000 epifluorescent microscope equipped with S Fluor ×40/1.3 objective. Fura-2 was excited by alternating 340 nm and 380 nm using a Polychrome IV monochromator (Till Photonics, Germany), and the probe emission light was filtered through a 520/20 bandpass filter and collected by a cooled CCD camera (Hamamatsu, Japan). The fluorescence signals were acquired and processed using MetaFluor software (Molecular Devices, USA). To quantify the differences in the amplitudes of Ca^2+^ transients, the ratio values were normalized using the formula ΔF/F0.

### qRT-PCR

Total RNA from WT and KI*-*STIM1^I115F^ myotubes was isolated using TRI-Reagent^®^ and reverse transcribed according to the manufacturer's instructions (Life Technologies). cDNA was then stored at −20°C until further use. qRT-PCRs were performed on 96-well plates (CFX96™ Real-Time PCR Detection Systems, Bio-Rad, Italy), in triplicate, and fluorescence intensity was assessed.

Primers used are listed in Table S1. Transcripts were normalized to the expression of ribosomal protein S18 mRNAs; for each gene, the threshold cycle (ΔCt) was calculated.

### Statistical analysis

The normality of data distributions was assessed using Shapiro–Wilk test and data are presented as mean±s.e.m. or median and interquartile range (IQR), according to distribution. Parametric [unpaired Student's *t*-test and one-way analysis of variance (ANOVA) followed by Tukey's post-hoc] or non-parametric (Mann–Whitney *U*-test and one-way Kruskal–Wallis H test followed by Dunn's post-hoc) statistical analyses were used. All statistical assessments were two-sided and *P*<0.05 was considered statistically significant. Statistical analyses were performed using Prism software (GraphPad Software, USA). Statistical significance was tested between the two mouse strains at the same age and was not tested for changes in time.

In calcium-imaging *in vitro* experiments, *n* represents the number of cells, and the number of independent experiments (defined as experiments performed on different days) is provided in the respective figure legends. For *in vivo* experiments, *n* represents the number of animals studied. In experiments involving histology, the figures shown are representative of at least three experiments (histologic coloration), performed on different experimental days on the tissue sections collected from all animals in each group.

This article is part of a special collection ‘A Guide to Using Neuromuscular Disease Models for Basic and Preclinical Studies’, which was launched in a dedicated issue guest edited by Annemieke Aartsma-Rus, Maaike van Putten and James Dowling. See related articles in this collection at http://dmm.biologists.org/collection/neuromuscular.

## Supplementary Material

Supplementary information

## References

[DMM041111C1] Abel A. M., Yang C., Thakar M. S. and Malarkannan S. (2018). Natural killer cells: development, maturation, and clinical utilization. *Front Immunol.* 9, 1-23. 10.3389/fimmu.2018.01869 30150991 PMC6099181

[DMM041111C2] Berna-Erro A., Redondo P. C. and Rosado J. A. (2012). Store-operated Ca(2+) entry. *Adv. Exp. Med. Biol.* 740, 349-382. 10.1007/978-94-007-2888-2_15 22453950

[DMM041111C3] Böhm J. and Laporte J. (2018). Gain-of-function mutations in STIM1 and ORAI1 causing tubular aggregate myopathy and Stormorken syndrome. *Cell Calcium* 76, 1-9. 10.1016/j.ceca.2018.07.008 30243034

[DMM041111C4] Böhm J., Chevessier F., Koch C., Peche G. A., Mora M., Morandi L., Pasanisi B., Moroni I., Tasca G., Fattori F. et al. (2014). Clinical, histological and genetic characterisation of patients with tubular aggregate myopathy caused by mutations in STIM1. *J. Med. Genet.* 51, 824-833. 10.1136/jmedgenet-2014-102623 25326555

[DMM041111C5] Dhanjal T. S., Pendaries C., Ross E. A., Larson M. K., Protty M. B., Buckley C. D. and Watson S. P. (2007). A novel role for PECAM-1 in megakaryocytokinesis and recovery of platelet counts in thrombocytopenic mice. *Blood* 109, 4237-4244. 10.1182/blood-2006-10-050740 17234740

[DMM041111C6] Fahrner M., Stadlbauer M., Muik M., Rathner P., Stathopulos P., Ikura M., Müller N. and Romanin C. (2018). A dual mechanism promotes switching of the Stormorken STIM1 R304W mutant into the activated state. *Nat. Commun.* 9, 825 10.1038/s41467-018-03062-w 29483506 PMC5827659

[DMM041111C7] Feske S. (2010). CRAC channelopathies. *Pflugers Arch.* 460, 417-435. 10.1007/s00424-009-0777-5 20111871 PMC2885504

[DMM041111C8] Feske S., Picar C. and Fischer A. (2010). Immunodeficiency due to mutation in ORAI1 and STIM1. *Clin. Immunol.* 135, 169-182. 10.1016/j.clim.2010.01.011 20189884 PMC2856745

[DMM041111C9] Gamage T. H., Gunnes G., Lee R. H., Louch W. E., Holmgren A., Bruton J. D., Lengle E., Kolstad T. R. S., Revold T., Amundsen S. S. et al. (2018). STIM1 R304W causes muscle degeneration and impaired platelet activation in mice. *Cell Calcium.* 76, 87-100. 10.1016/j.ceca.2018.10.001 30390422 PMC6481308

[DMM041111C10] Garibaldi M., Fattori F., Riva B., Labasse C., Brochier G., Ottaviani P., Sacconi S., Vizzaccaro E., Laschena F., Romero N. et al. (2016). A novel gain-of-function mutation in ORAI1 causes late-onset Tubular Aggregate Myopathy and congenital miosis. *Clin. Genet.* 91, 780-786. 10.1111/cge.12888 27882542

[DMM041111C11] Goonasekera S. A., Davis J., Kwong J. Q., Accornero F., Wei-LaPierre L., Sargent M. A., Dirksen R. T. and Molkentin J. D. (2014). Enhanced Ca2+ influx from STIM1–Orai1 induces muscle pathology in mouse models of muscular dystrophy. *Hum. Mol. Genet.* 23, 3706-3715. 10.1093/hmg/ddu079 24556214 PMC4065147

[DMM041111C12] Grosse J., Braun A., Varga-Szabo D., Beyersdorf N., Schneider B., Zeitlmann L., Hanke P., Schropp P., Mühlstedt S., Zorn C. et al. (2007). An EF hand mutation in Stim1 causes premature platelet activation and bleeding in mice. *J. Clin. Invest.* 117, 3540-3550. 10.1172/JCI32312 17965774 PMC2040319

[DMM041111C13] Harris E., Hudson J., Marsh J., Marini Bettolo C., Neri M., Ferlini A., Bushby K., Lochmüller H., Straub B. and Barresi R. (2015). A novel STIM1 mutation at p.340 causes tubular aggregate myopathy with miosis without additional features of Stormorken syndrome. *Neuromuscul. Disord* 25, S289 10.1016/j.nmd.2015.06.369

[DMM041111C14] Lacruz R. S. and Feske S. (2015). Diseases caused by mutations in ORAI1 and STIM1. *Ann. NY Acad. Sci.* 1356, 45-79. 10.1111/nyas.12938 26469693 PMC4692058

[DMM041111C15] Markello T., Chen D., Kwan J. Y., Horkayne-Szakaly I., Morrison A., Simakova O., Maric I., Lozier J., Cullinane A. R., Kilo T. et al. (2015). York platelet syndrome is a CRAC channelopathy due to gain-of-function mutations in STIM1. *Mol. Genet. Metab.* 114, 474-482. 10.1016/j.ymgme.2014.12.307 25577287 PMC4355183

[DMM041111C16] Misceo D., Holmgren A., Louch W. E., Holme P. A., Mizobuchi M., Morales R. J., De Paula A. M., Stray-Pedersen A., Lyle R., Dalhus B. et al. (2014). A dominant STIM1 mutation causes Stormorken syndrome. *Hum. Mutat.* 35, 556-564. 10.1002/humu.22544 24619930

[DMM041111C17] Nesin V., Wiley G., Kousi M., Ong E.-C., Lehmann T., Nicholl D. J., Suri M., Shahrizaila N., Katsanis N., Gaffney P. M. et al. (2014). Activating mutations in STIM1 and ORAI1 cause overlapping syndromes of tubular myopathy and congenital miosis. *Proc. Natl. Acad. Sci. USA* 111, 4197-4202. 10.1073/pnas.1312520111 24591628 PMC3964084

[DMM041111C18] Noury J.-B., Böhm J., Peche G. A., Guyant-Marechal L., Bedat-Millet A.-L., Chiche L., Carlier R.-Y., Malfatti E., Romero N. B. and Stojkovic T. (2017). Tubular aggregate myopathy with features of Stormorken disease due to a new STIM1 mutation. *Neuromuscul. Disord.* 27, 78-82. 10.1016/j.nmd.2016.10.006 27876257

[DMM041111C19] Okuma H., Saito F., Mitsui J., Hara Y., Hatanaka Y., Ikeda M., Shimizu T., Matsumura K., Shimizu J., Tsuji S. et al. (2016). Tubular aggregate myopathy caused by a novel mutation in the cytoplasmic domain of STIM1. *Neurol Genet.* 2, e50 10.1212/NXG.0000000000000050 27066587 PMC4817897

[DMM041111C20] Ong H. L. and Ambudkar I. S. (2015). Molecular determinants of TRPC1 regulation within ER-PM junctions. *Cell Calcium* 58, 376-386. 10.1016/j.ceca.2015.03.008 25922260

[DMM041111C21] Putney J. W. (2011). Origins of the concept of store-operated calcium entry. *Front. Biosci.* 3, 980-984. 10.2741/s202 PMC3931420 21622247

[DMM041111C22] Schiaffino S. (2012). Tubular aggregates in skeletal muscle: just a special type of protein aggregates? *Neuromuscul. Disord.* 22, 199-207. 10.1016/j.nmd.2011.10.005 22154366

[DMM041111C23] Shiotsuki H., Yoshimi K., Shimo Y., Funayama M., Takamatsu Y., Ikeda K., Takahashi R., Kitazawa S. and Hattor N. (2010). A rotarod test for evaluation of motor skill learning. *J. Neurosci. Methods* 189, 180-185. 10.1016/j.jneumeth.2010.03.026 20359499

[DMM041111C24] Silva-Rojas R., Treves S., Jacobs H., Kessler P., Messaddeq N., Laporte J. and Böhm J. (2018). STIM1 overactivation generates a multi-systemic phenotype affecting skeletal muscle, spleen, eye, skin, bones, and the immune system in mice. *Hum. Mol. Genet.* 28, 1579-1593. 10.1093/hmg/ddy446 30576443

[DMM041111C25] Stormorken H., Holmsen H., Sund R., Sakariassen K. S., Hovig T., Jellum E. and Solum O. (1995). Studies on the haemostatic defect in a complicated syndrome. An inverse Scott syndrome platelet membrane abnormality. *Thromb. Haemost.* 74, 1244-1251. 10.1055/s-0038-1649920 8607103

[DMM041111C26] Walter M. C., Rossius M., Zitzelsberger M., Vorgerd M., Müller-Felber W., Ertl-Wagner B., Zhang Y., Brinkmeier H., Senderek J. and Schoser B. (2015). 50 years to diagnosis: autosomal dominant tubular aggregate myopathy caused by a novel STIM1 mutation. *Neuromuscul. Disord.* 25, 577-584. 10.1016/j.nmd.2015.04.005 25953320

