# Supplementary material for: A luminal EF-hand mutation in STIM1 in mice causes the clinical hallmarks of tubular aggregate myopathy
Source: Dis Model Mech. 2019 Dec 3;13(2):dmm041111. doi: 10.1242/dmm.041111 (PMC6906633; doi:10.1242/dmm.041111)
Supplement: Supplementary information [file dmm-13-041111-s1.pdf]

Figure S1

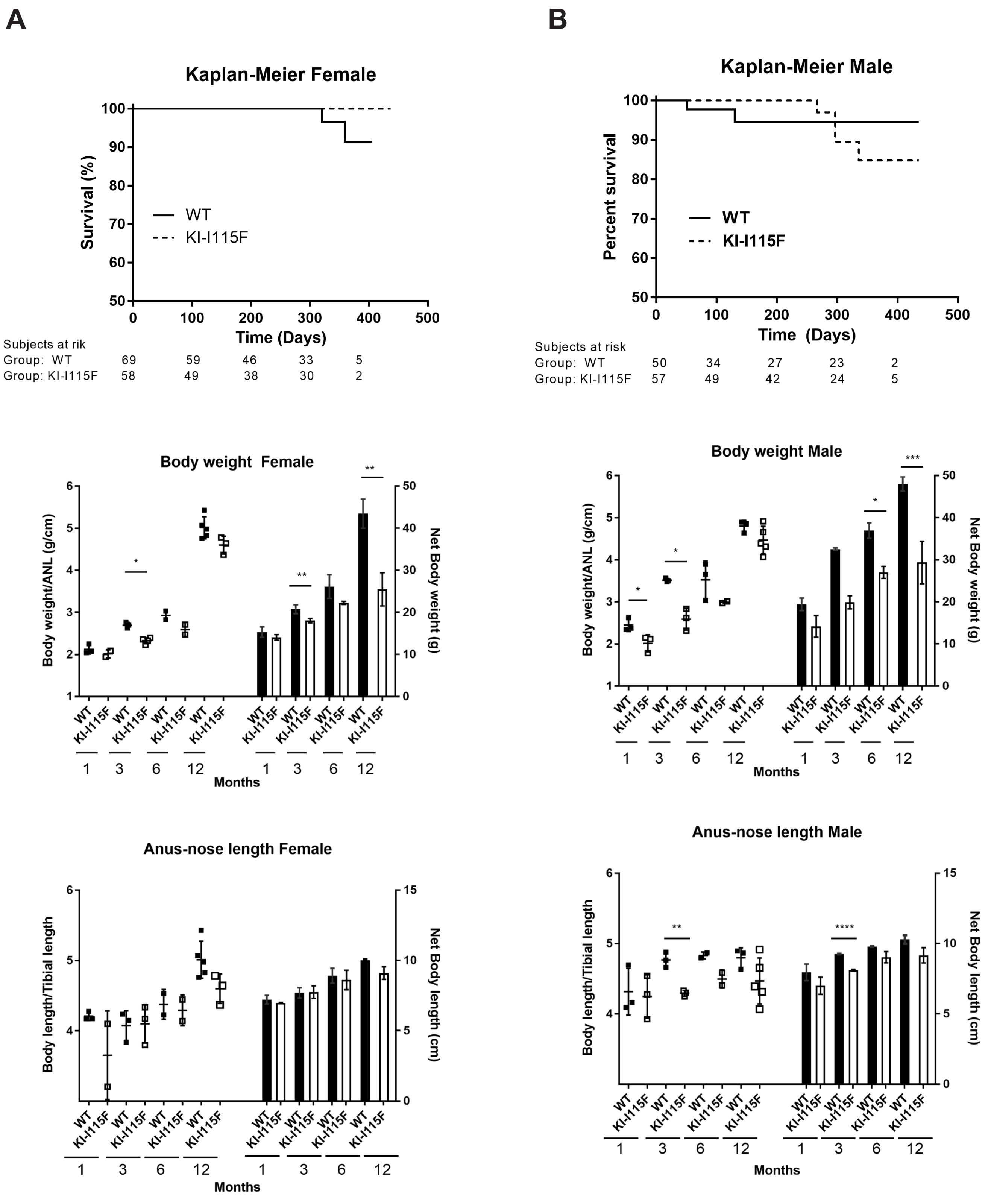

Figure S1. Characterization of KI-STIM1I115F mice in female and male animals. (**A**) Sub-analysis of data for female animals. Top panel shows Kaplan-Meier for survival. Middle panel shows body weight at 1 (WT n=3, KI-I115F n=2), 3 (WT n=3, KI-I115F n=4), 6 (WT n=2, KI-I115F n=2) and 12 (WT n=5, KI-I115F n=3) months of age. Scatter plots and histograms show the means ± S.E.M of the indicated number of mice. Unpaired Student t-test with Welch's correction.* P=0.0302, ** P≤0.0033 versus WT. Bottom panel shows body length at 1 (WT n=3, KI-I115F n=2), 3 (WT n=3, KI-I115F n=3), 6 (WT n=2, KI-I115F n=2) and 12 (WT n=4, KI-I115F n=3). Scatter plots and histograms show the means ± S.E.M of the indicated number of mice. Unpaired Student t-test with Welch's correction versus WT. (**B**) Sub-analysis of data for male animals; Top panel shows Kaplan-Meier for survival. Middle panel shows body weight at 1 (WT n=3, KI-I115F n=3), 3 (WT n=3, KI-I115F n=3), 6 (WT n=2, KI-I115F n=2) and 12 (WT n=3 KI-I115F n=5) months of age. Scatter plots and histograms show the means ± S.E.M of the indicated number of mice. Unpaired Student t-test with Welch's correction.* P≤0.0455, *** P=0.0005 versus WT. Bottom panel shows body length at 1 (WT n=3, KI-I115F n=3), 3 (WT n=3, KI-I115F n=3), 6 (WT n=2, KI-I115F n=2) and 12 (WT n=3, KI-I115F n=5) months of age. Scatter plots and histograms show the means ± S.E.M of the indicated number of mice. Unpaired Student t-test with Welch's correction. ** P=0.0093, **** $P<10^{-4}$ versus WT.

Figure S2

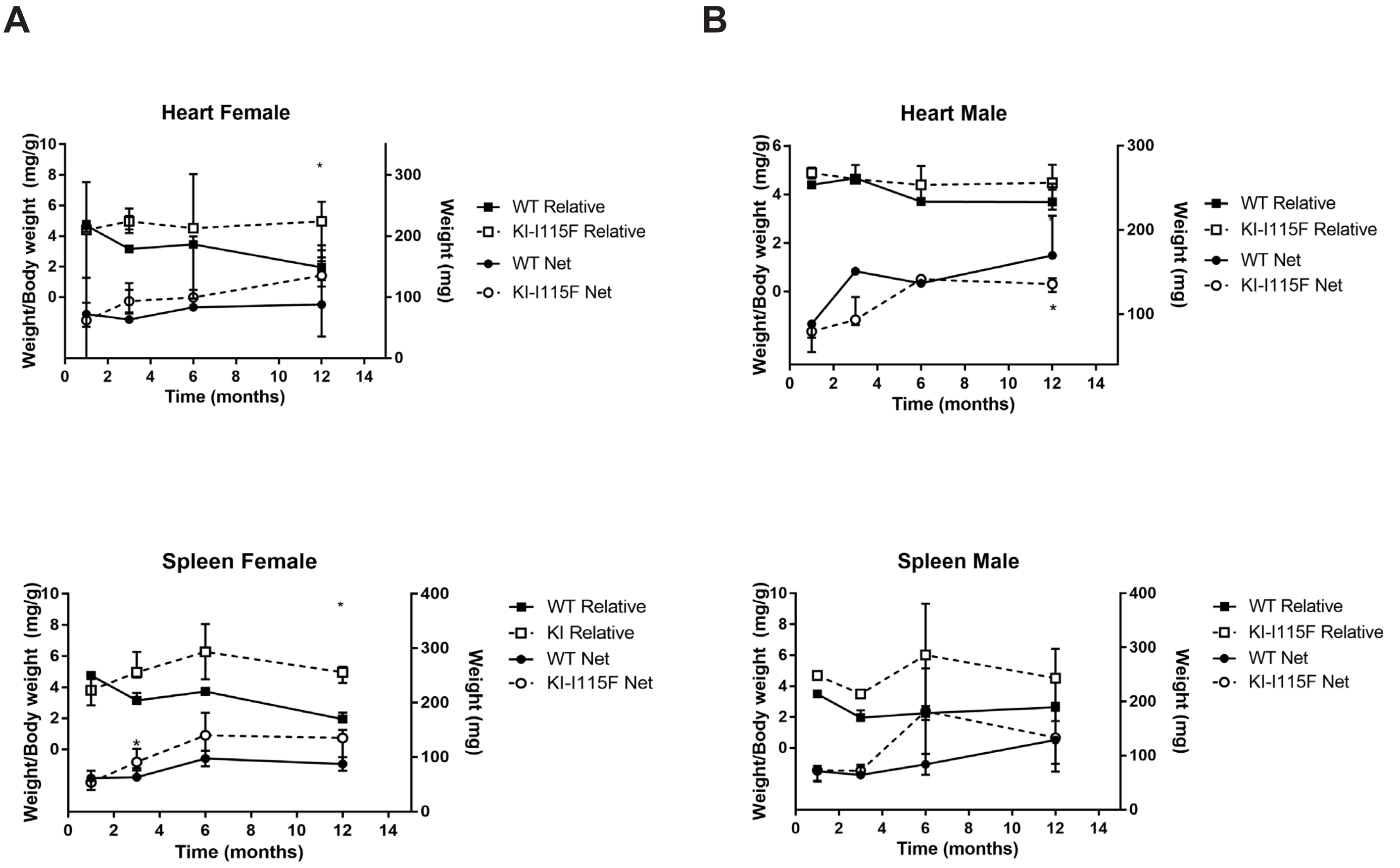

Figure S2. Sub-analysis of heart and spleen size in female (A) and male (B) animals. (**A**) Top panel represents heart weight and bottom panel represents spleen weight at 1 (WT n=3, KI-I115F n=2), 3 (WT n=3, KI-I115F n=3), 6 (WT n=2, KI-I115F n=2) and 12 (WT n=5, KI-I115F n=3) months of age. Graph shows median and IQR of heart weight/body weight, or heart net weight. Mann-Whitney U test. * P=0.0357 versus WT. (**B**) Heart weight (top panel) and spleen weight (bottom panel) at 1 (WT n=3, KI-I115F n=3), 3 (WT n=3, KI-I115F n=3), 6 (WT n=2, KI-I115F n=2) and 12 (WT n=3, KI-I115F n=5) months of age. Graph shows median and IQR of heart weight/body weight, or heart net weight. Mann-Whitney U test. * P=0.0357 versus WT.

Disease Models & Mechanisms • Supplementary information

Figure S3

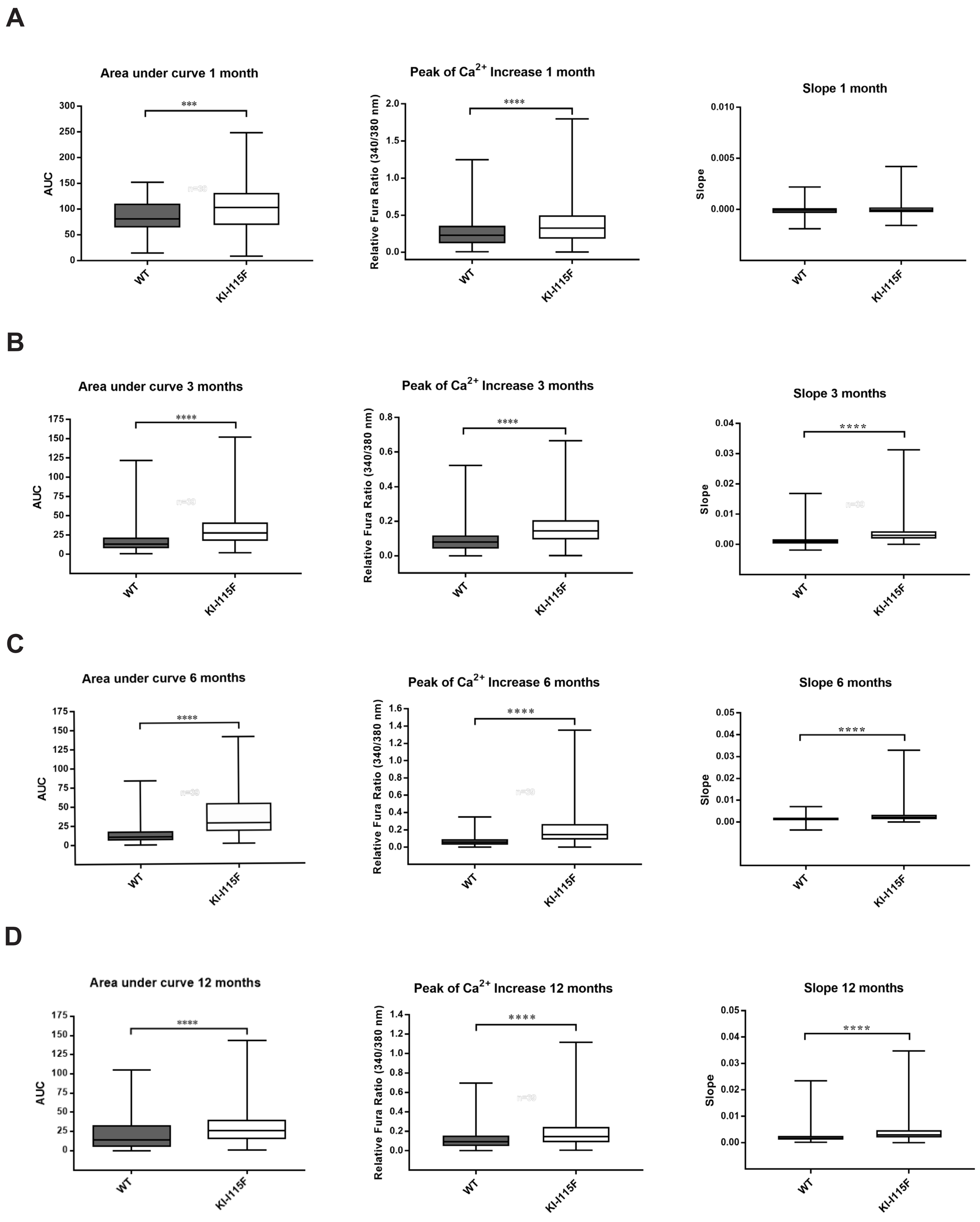

Figure S3. SOCE alterations in myotubes. Evaluation of the area under the curve (AUC), the peak amplitude, and the slope of the Ca^{2+}-rise in WT and KI-I115F at 1 (**A**), 3 (**B**), 6 (**C**) and 12 (**D**) months of age. Traces are the average of at least 180 myotubes from 6-well plates on two different experimental days. At all time-point, the experiments were performed on 4 different individual mice two males and two females per strain. Graph shows median and IQR of AUC, peak amplitude and slope of the Ca^{2+}-rise. Mann-Whitney U test. AUC 1 months *** P=0.0005, peak amplitude 1 months **** P<0.0001 versus WT; AUC, peak amplitude and slope of Ca^{2+}-rise 3 months **** P<0.0001 versus WT; AUC, peak amplitude and slope of Ca^{2+}-rise 6 months **** P<0.0001 versus WT; AUC, peak amplitude and slope of Ca^{2+}-rise 12 months **** P<0.0001 versus WT.

Figure S4

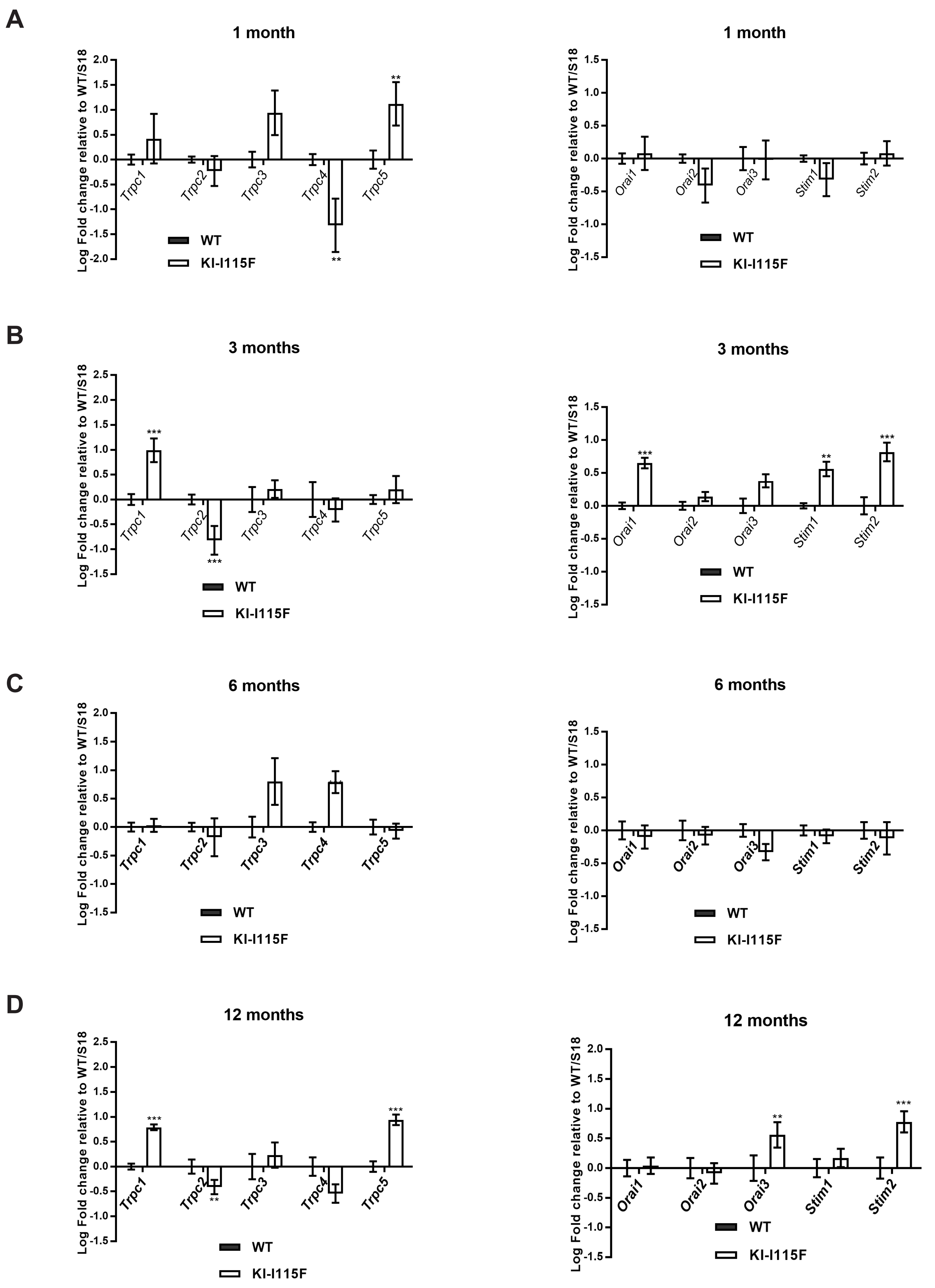

Figure S4. Gene expression alterations in muscle. RT-PCR of SOCE genes in WT and KI-I115F at 1 (**A**), 3 (**B**), 6 (**C**) and 12 (**D**) months of age. Values represent mean ± S.E.M and are expressed as ΔC(t) of genes/S18 of four independent cultures. Unpaired two-tailed Student's t-test ** P≤0.00456, *** P≤0.000351 versus WT.

Figure S5

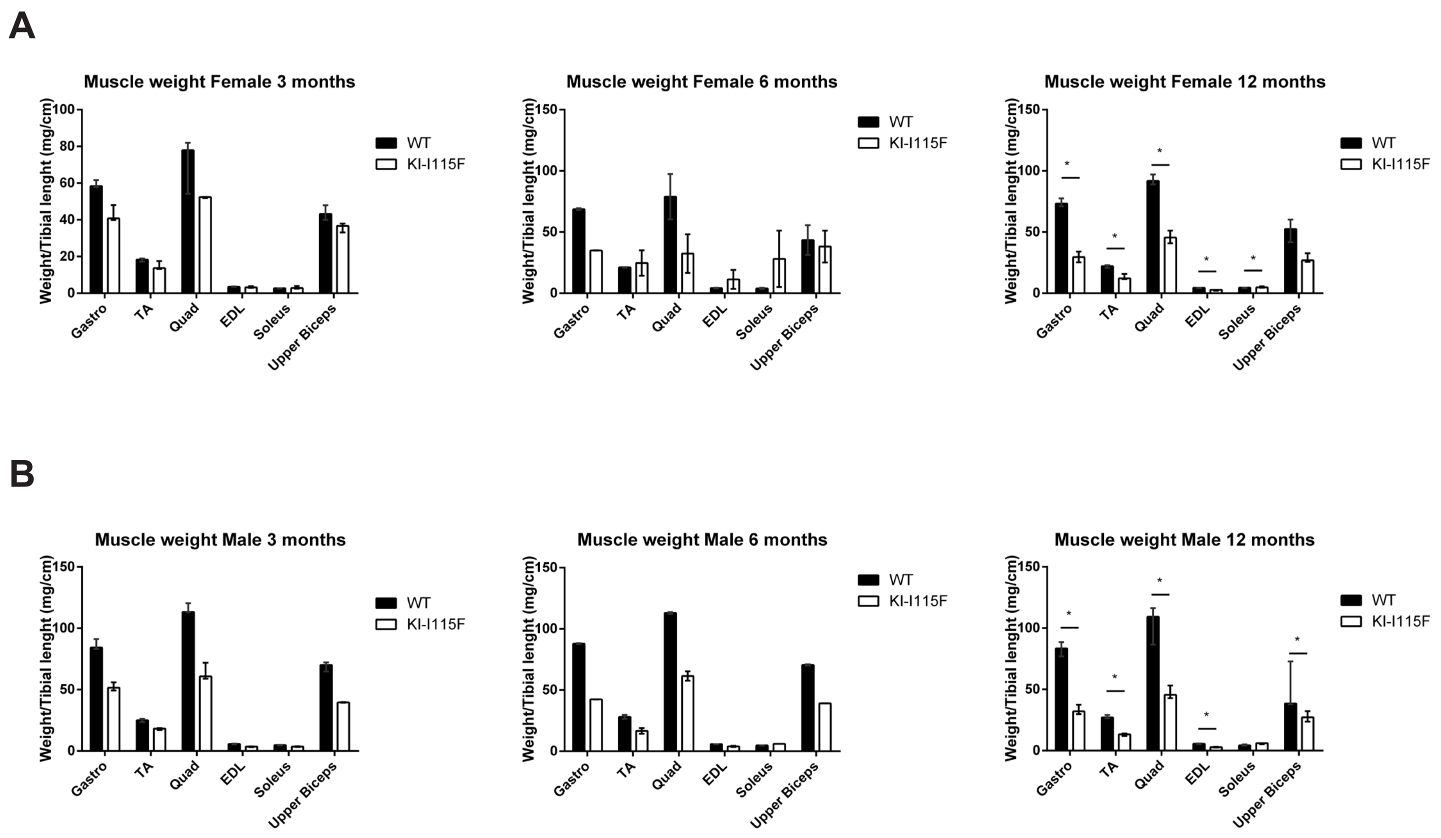

Figure S5. Sub-analysis of muscle growth and damage in female (A) and male (B) animals. (**A**) Muscle weight at 3 (WT n=3, KI-I115F n=3), 6 (WT n=2, KI-I115F n=2) and 12 months (WT n=5, KI-115F n=3) of age. (Gastro: gastrocnemius, TA: tibialis anterior, Quad: quadriceps, EDL: extensor digitorum longus). Histograms show median and IQR of muscle weights. Mann-Whitney U test. * P=0.0357 versus WT. (**B**) Muscle weight at 3 (WT n=3, KI-I115F n=3), 6 (WT n=2, KI-I115F n=2) and 12 months (WT n=3, KI-I115F n=5) of age. (Gastro: gastrocnemius, TA: tibialis anterior, Quad: quadriceps, EDL: extensor digitorum longus). Histograms show median and IQR of muscle weights. Mann-Whitney U test. * P=0.0357 versus WT.

Figure S6

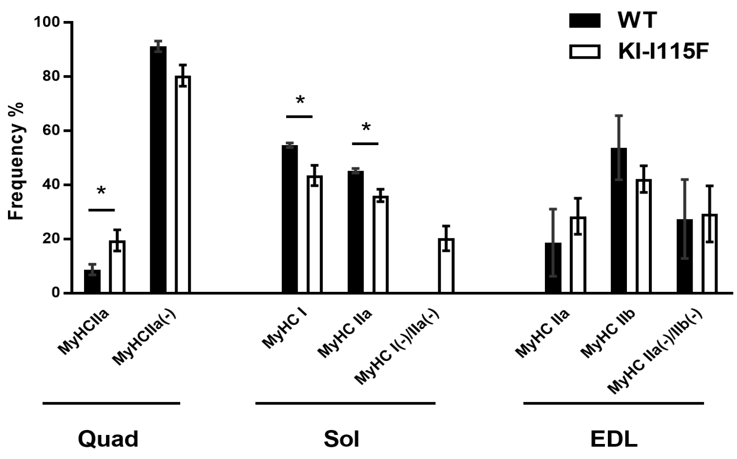

Figure S6. Muscle fiber frequency distribution in 12 months old WT and KI-I115F mice. Myosin heavy-chain (MyHC) immunofluorescence fiber staining (n=3) on quadriceps (Quad) for type IIa fibers (oxidative, fatigue resistant), on soleus (Sol) for type I and IIa fibers and on extensor digitorum longus (EDL) for type IIa and IIb (glycolytic) fibers. Negative myofibers for quadriceps represent type IIx and IIb fibers (MyHC IIa(-)) for soleus type IIx and IIb (MyHC I(-)/IIa(-)) and for EDL type IIx (MyHC IIa(-)/IIb(-)). The frequency is calculated above the total number of muscle fibers. Histograms show the means ± S.E.M of the indicated number of mice. Unpaired Student t-test with Welch’s correction.* P≤0.042 versus WT.

Figure S7

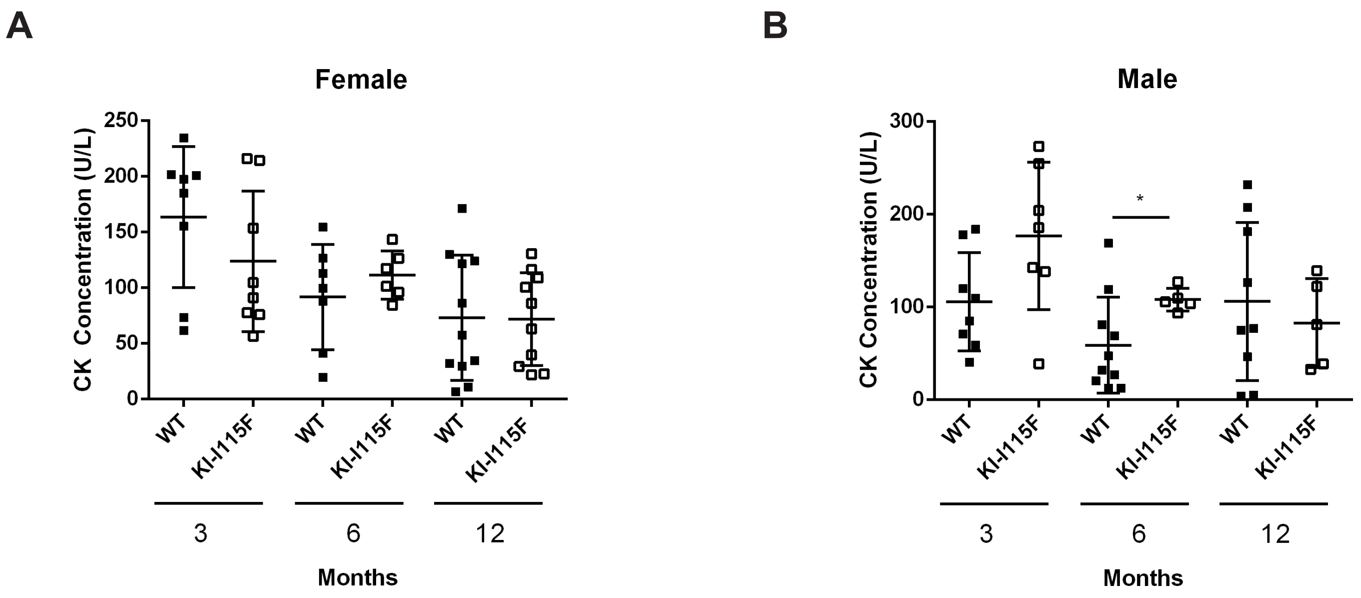

Figure S7. Sub-analysis of Creatine Kinase plasma levels in female (A) and male (B) animals.
(**A**) Scatter plots and histograms show the means ± S.E.M at 3 months (WT n=8, KI-I115F n=8), 6 months (WT n=7, KI-I115F n=6), 12 months (WT n=11, KI-I115F n=10) of age. Unpaired Student t-test with Welch's correction versus WT. (**B**) Scatter plots and histograms show the means ± S.E.M at 3 months (WT n=8, KI-I115F n=7), 6 months (WT n=10, KI-I115F n=5), 12 months (WT n=9, KI-I115F n=5) of age. Unpaired Student t-test with Welch's correction. * P=0.016 versus WT.

Disease Models & Mechanisms • Supplementary information

Figure S8

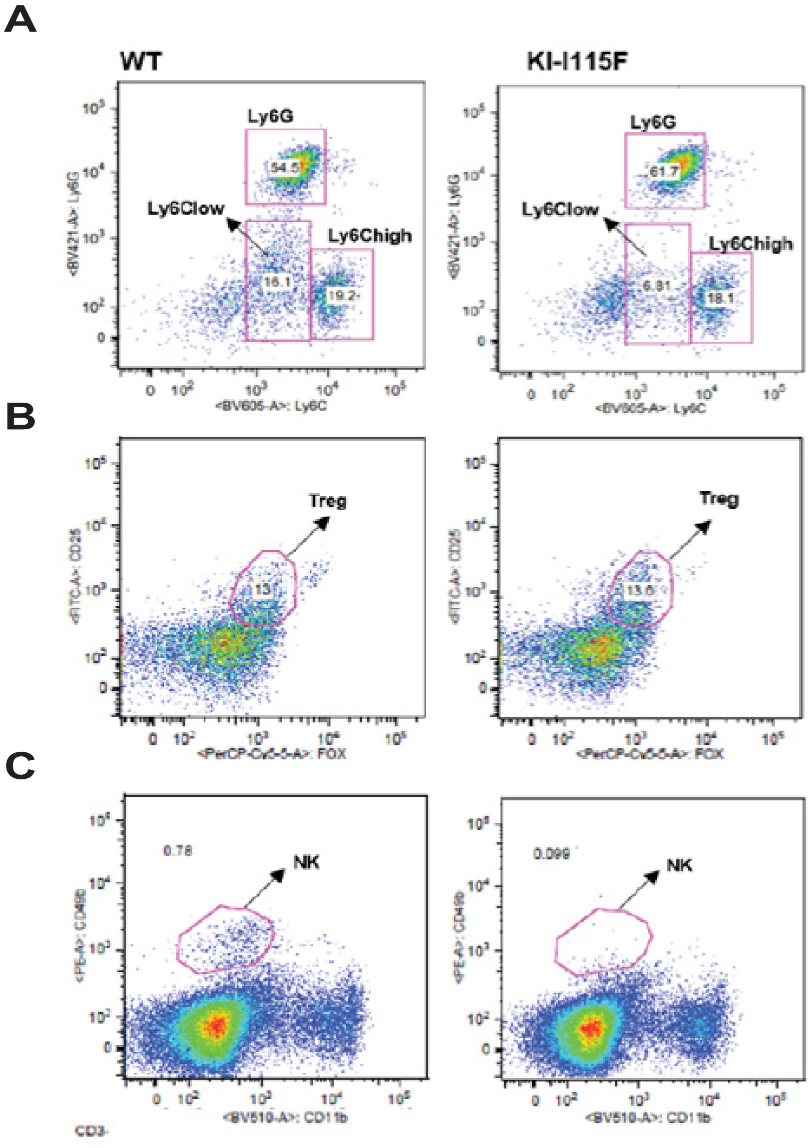

Figure S8. Representative gating strategies in FACS analysis
(**A**) Evaluation of monocytes and granulocytes in mouse blood using anti-Ly6C and anti-Ly6G antibodies. (**B**) Evaluation of Treg cells in mouse spleen determined by anti-FOXp3 and anti-CD25 antibodies. (**C**) Evaluation of NK cells in mouse spleen using anti-CD11b and anti-CD49b antibodies. Please refer to the Material and method section for further details.

Disease Models & Mechanisms • Supplementary information

Table S1

Gene Symbol	Forward Primers	Reverse Primers
Trpc1	5'- GGACAGCCTCAGACATTCCA -3'	5'- CGGGCTAGCTCTTCATAATCA -3'
Trpc2	5'- AGAGGCAGAGCTGGAGTTCA -3'	5'- GGGGAATTTGAGCTTTTTGTT -3'
Trpc3	5'- GATCAATGTCTACAAGGGACT -3'	5'- TGCATTGCATGGAGAGTTTC -3'
Trpc4	5'- ACGCCATCAGAAAAGAGGTG -3'	5'- CCAAGATGATGGGTGTGATG -3'
Trpc5	5'- GAGGTGGTAGGAGCTGTGGA -3'	5'- TGCCAACATAATGGGAGTGA -3'
Stim1	5'- TCTGAAGAGTCTACCGAAGCAG -3'	5'- TGGTAATTGAGGTCTTCCCTTAG -3'
Stim2	5'- GACGAATGCGATCTGGTG -3'	5'- TTCAGTGAAGCAAGGTGGACT -3'
Orai1	5'- CCTGGCGCAAGCTCTACTTA -3'	5'- TGCAGGCACTAAAGACGATC -3'
Orai2	5'- CACTGTCCTGGAGGAAGCTC -3'	5'- GGGCTGAGGTACTGGTACTT -3'
Orai3	5'-GAACCCGGAGGTGGACAG -3'	5'- GCTGGAGGCTTTGAGCATTAG -3'

Table S1. List of primers used

Disease Models & Mechanisms • Supplementary information
